# Identification and Regulation of Melatonin Biosynthetic Genes in Sweet Pepper During Ripening and Melatonin Treatment

**DOI:** 10.3390/antiox15040503

**Published:** 2026-04-17

**Authors:** Jorge Taboada, Lourdes Sánchez-Moreno, José M. Palma, Francisco J. Corpas

**Affiliations:** Group of Antioxidants, Free Radicals and Nitric Oxide in Biotechnology, Food and Agriculture, Department of Stress, Development and Signaling in Plants, Estación Experimental del Zaidín, Spanish National Research Council (CSIC), C/Profesor Albareda 1, 18008 Granada, Spain; jorge.taboada@eez.csic.es (J.T.); lourdes.sanchez@eez.csic.es (L.S.-M.); josemanuel.palma@eez.csic.es (J.M.P.)

**Keywords:** *Capsicum annuum*, melatonin biosynthesis, pepper fruit, ripening, postharvest physiology, RNAseq

## Abstract

Since its discovery in higher plants, melatonin has attracted considerable attention for its antioxidant properties and its diverse roles in plant physiology and stress responses. However, its biosynthetic pathway remains only partially elucidated, particularly in horticultural crops of economic and nutritional importance, such as pepper (*Capsicum annuum* L.) fruits. In our previous work, we identified five genes encoding tryptophan decarboxylase (TDC), the first enzyme in the melatonin biosynthetic pathway in pepper. The present study expands on this by identifying and characterizing additional genes encoding enzymes involved in subsequent steps of the pathway, including four *tryptamine 5-hydroxylase* (*T5H*) genes, two *serotonin N-acetyltransferase* (*SNAT*) genes, three *N-acetylserotonin O-methyltransferase* (*ASMT*) genes, two *caffeic acid O-methyltransferase* (*COMT*) genes, and one *N-acetylserotonin deacetylase* (*ASDAC*) gene, representing a total of twelve newly identified genes. We further examined their expression in sweet pepper fruits and found that only nine of the identified genes are expressed in the fruit, with generally higher transcript levels during the unripe stages. Melatonin quantification in the California-type ‘Masami’ cultivar using UPLC with fluorescence detection (FD) revealed concentrations of 623 ng melatonin·g^−1^ dry weight (DW) in green fruits and 431 ng melatonin·g^−1^ DW in red fruits, consistent with the higher expression of melatonin biosynthetic genes in unripe fruit. Expression analysis of these genes by means of RNA-seq revealed differential modulation in response to exogenous melatonin treatments (20, 50, and 100 µM). To our knowledge, this is the first report demonstrating that exogenous melatonin regulates the expression of genes involved in its own biosynthetic pathway in sweet pepper fruits. Notably, treatment with 100 µM melatonin delayed ripening in these non-climacteric fruits, highlighting its potential biotechnological application for controlling fruit ripening and improving postharvest management.

## 1. Introduction

Since its identification in 1995 as a molecule synthesized by plant cells, melatonin (5-methoxy-N-acetyltryptamine) has attracted substantial research interest. Consequently, the number of related publications in higher plants has increased sharply over the past decade, rising from 72 in 2015 to 334 in 2025, according to PubMed, with a total of 1835 publications published over the last ten years. In addition to its well-established antioxidant properties, melatonin plays a role in regulating diverse physiological processes and mediating plant responses to adverse environmental conditions [1,2,3,4,5,6]. Furthermore, exogenous application of melatonin has been shown to confer beneficial effects across a wide range of plant species [7,8,9,10,11]. One of the most important areas of current research focuses on elucidating the melatonin biosynthetic pathway, which appears to be more complex in plants than in animals. To date, this pathway has been most extensively characterized in rice (*Oryza sativa*) and *Arabidopsis thaliana*; however, significant knowledge gaps remain for other plant species. In particular, the genes involved in melatonin biosynthesis in pepper (*Capsicum annuum*) have not yet been fully identified.

As mentioned, the melatonin biosynthesis pathway is well established in arabidopsis and rice, where key enzymes such as *tryptophan decarboxylase* (*TDC*), *serotonin N-acetyltransferase* (*SNAT*), *caffeic acid O-methyltransferase* (*COMT*), and *N-acetylserotonin O-methyltransferase* (*ASMT*) have been functionally characterized and their expression patterns clearly defined [12,13,14,15,16,17]. In tomato, although homologous genes have been identified through genome assembly [18], the pathway remains less comprehensively understood. Nevertheless, the generation of mutant lines has begun to provide important insights into melatonin biosynthesis and its physiological roles in tomato plants [19,20,21,22,23]. In contrast, knowledge of this pathway in pepper is still very limited, with most available studies focusing primarily on the effects of exogenous melatonin rather [24,25,26] than on the molecular characterization of endogenous biosynthetic genes.

Peppers (*Capsicum* spp.), members of the Solanaceae family, are globally significant crops valued for their high nutritional and economic importance. They are notable for being rich in antioxidants, with vitamin C as the most abundant bioactive compound [27,28] as well as capsaicin and derived compounds in hot pepper varieties [29,30]. During fruit ripening, peppers undergo significant molecular changes (transcriptomics, proteomics, and metabolomics remodeling). The most evident change is the shift in color from green to varieties like red, yellow, orange, or purple due to the accumulation of carotenoids [31]. Recent research indicates that ripening also involves nitro-oxidative stress, leading to alterations in the metabolism of reactive oxygen and nitrogen species (ROS and RNS) [32,33,34,35,36]. Consequently, antioxidant enzymes (e.g., ascorbate peroxidase, catalase) are dynamically regulated throughout the ripening process [37,38,39].

In a previous work, we identified the genes encoding the first enzyme of the melatonin biosynthesis pathway in pepper, specifically those encoding tryptophan decarboxylase (TDC), which comprised a total of five genes [40]. The present study, using California-type sweet pepper fruits, builds upon this foundation by identifying and characterizing additional genes encoding enzymes with *SNAT*, *ASMT*, *COMT*, and *N-acetylserotonin deacetylase* (*ASDAC*) activities, representing a total of twelve genes. Additionally, we quantified melatonin content during ripening and further examined the expression of the identified genes by means of RNA-seq analysis during sweet pepper fruit ripening and under exogenous melatonin treatment.

## 2. Materials and Methods

### 2.1. Plant Material

Sweet pepper (*C. annuum* L., cv. Masami, California type) fruits were collected between January and February 2023 from plants cultivated in plastic-covered greenhouses (Syngenta Seeds, Ltd., Roquetas de Mar/El Ejido, Almería, Spain). During the growing period, the mean outdoor temperature was 11 °C and solar radiation averaged 13 MJ·m^−2^ day^−1^, based on records from the La Mojonera Meteorological Station (Junta de Andalucía, Spain; UTM X: 526472.0, Y: 4071536.0; 36°47′19″ N, 02°42′11″ W; 142 m above sea level). Greenhouse temperatures were generally 5–7 °C higher than external values. Fruits free of visible defects were harvested at three developmental stages: green (immature), breakpoint (BP), and red (fully ripe). After harvest, the fruits were placed in black plastic bags and transported to the laboratory. There, the fruits were washed with distilled water, gently dried with paper towels, and stored at 7 ± 1 °C for 12 h. The following day, fruits at the BP stage were selected for melatonin treatments. Green and red fruits were cut into approximately 5 mm^2^ cubes, immediately frozen in liquid nitrogen, and stored at −80 °C until further analysis.

### 2.2. Identification of the Genes Encoding the Enzymes Involved in Melatonin Biosynthesis in Pepper and Their Chromosomal Location

Pepper homologs of melatonin biosynthetic genes were identified using the *C. annuum* genome and proteome (BioProject: PRJNA814299, UCD10Xv1.1; [41]) through BLASTp (https://blast.ncbi.nlm.nih.gov) searches against reference proteins from rice (*Oryza sativa*) and *Arabidopsis thaliana*. For the identification of T5H-coding genes (*CaT5H*s), OsT5H (NP_001410381.1) [12] was used, applying a specific criterion to ensure selectivity and prevent interference from closely related cytochrome P450s. These criteria required ≥40% sequence identity and ≥90% query coverage, with the E-value threshold set to ≤ 1 × 10^−100^. The presence of the conserved cytochrome P450 domain (PF00067) and the characteristic P450 site (IPR017972) was subsequently verified using InterProScan 5 [42]. Candidate *CaSNAT*s were identified using OsSNAT (NP_001389503.1) [14] with thresholds of ≥55% identity, ≥70% coverage and an E-value ≤ 1 × 10^−50^, all containing the General Control Non-repressible 5 (GCN5)-related *N-acetyltransferase* (GNAT) domain (PF00583). *CaASMT*s were detected based on similarity to *AtASMT* (NP_195242.1) [13], requiring ≥48% identity, ≥93% coverage and an E-value ≤ 1 × 10^−100^ as well as the presence of both the *O-methyltransferase* (PF00891) and dimerization (PF08100) domains. *CaCOMT*s were identified using AtCOMT (NP_200227.1) [15] with ≥70% identity, ≥96% coverage and an E-value of 0 and similarly exhibited PF00891 and PF08100 domains. Finally, *CaASDAC* was identified using OsASDAC (XP_015618357.1) and AtASDAC (NP_567921.1) [43], with ≥70% identity, ≥90% coverage, and an E-value of 0 and sharing the histone deacetylase domain (PF00850).

Chromosomal mapping was carried out using MG2C v2.1 [44]. Phylogenetic analyses were performed with CLUSTALW [45] using the maximum similarity method and 250 bootstrap replicates, and the resulting trees were visualized with Evolview v3 [46].

### 2.3. Analysis of Gene Structure and Regulatory Elements

To examine the structural organization of the genes encoding the enzymes involved in melatonin biosynthesis, information on exon–intron composition and the 5′ and 3′ UTRs was retrieved from the NCBI Nucleotide database (https://www.ncbi.nlm.nih.gov/nucleotide/, accessed 6 March 2024). In addition, the regulatory elements potentially involved in the transcriptional control of these genes were analyzed. For this purpose, the 2000 base pairs (bp) upstream of the transcription start site were extracted, as this region typically contains key regulatory motifs. These promoter sequences were obtained from the NCBI Nucleotide database, and cis-acting regulatory elements were identified using the PlantCARE tool (https://bioinformatics.psb.ugent.be/webtools/plantcare/html/, accessed on 14 October 2025). The resulting regulatory elements were then visualized with the “HeatMap” function in TBTools v2.069 [47].

### 2.4. Analysis of Localization and Protein Characteristics

Subcellular localization of the enzymes involved in melatonin biosynthesis was predicted using the WoLF PSORT online tool (https://wolfpsort.hgc.jp/, accessed on 21 October 2025) [48], based on analyses of their amino acid sequences. Additional protein features, including molecular weight and isoelectric point, were also evaluated. These characteristics were predicted from the amino acid sequences of each protein using the Compute pI/MW tool available on the ExPASy server (https://www.expasy.org/, accessed on 21 October 2025) [49].

### 2.5. Phylogenetic Analysis

For the phylogenetic analysis, orthologous proteins from a range of plant species were retrieved, and sequence alignments were performed using the CLUSTALW tool provided by EMBL-EBI (https://www.ebi.ac.uk/jdispatcher/msa/clustalo, accessed on 4 November 2025). The sequences included in this analysis are listed in the Supplementary Tables S1–S5 [17,50,51,52,53,54,55,56,57,58,59,60,61,62,63,64,65,66,67,68,69,70,71,72,73,74,75,76,77,78,79]. Phylogenetic trees were subsequently constructed in MEGA11 [80] using the maximum similarity method and the program’s default parameters.

### 2.6. Exogenous Melatonin (20, 50, and 100 µM) Treatment of California Sweet Pepper Fruits Masami Cultivar

**Figure 1:**
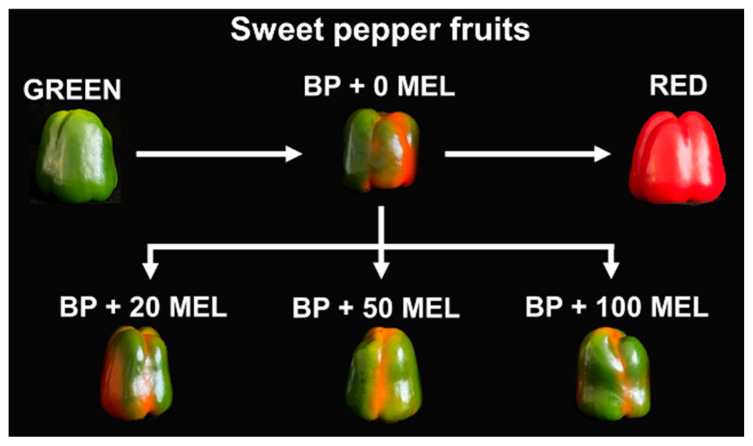
Schematic representation of the experimental design for the exogenous application of melatonin (MEL) to sweet pepper fruits at the breaking point (BP) stage, using concentrations of 0, 20, 50, and 100 µM.

Pepper fruits at the breaking point (BP) stage were divided into four groups of five fruits each. Each group was photographed on both the side showing the highest degree of ripeness (Side A) and the opposite side (Side B), and then sprayed with 25 mL of melatonin solutions at the indicated concentrations (20, 50, and 100 µM). The last group was sprayed with the same volume of distilled water as a treatment control. They were then allowed to air dry on a tray and left at room temperature until the third day, when they were photographed and treated again with either melatonin or distilled water. On the fourth day, the different groups of peppers were photographed again, and the decision was made to end the experiment because some fruits had reached full ripeness, that is, were completely red. The overall experimental design is illustrated in Figure 1.

### 2.7. RNA Extraction, Preparation of the Gene Library for RNA Sequencing of Pepper Fruits, and KEGG Pathway Enrichment Analysis

Total RNA was extracted from pepper fruits at different ripening stages and melatonin treatments using a two-step method combining Trizol^®^ Reagent and the RNAeasy Plant Mini Kit (Qiagen, Barcelona, Spain), as previously described [81]. This approach enhances RNA quality, making it particularly suitable for RNA-seq analyses. For each stage of fruit ripening and each melatonin treatment, three RNA-seq assays were conducted. Sample quality was confirmed via spectrophotometric quantification (NanoDrop, Thermo Fisher, Madrid, Spain, measuring 260/280 nm and 260/230 nm ratios) and agarose gel electrophoresis (visualized on a ChemiDoc-It^®^TS3 Imaging System, BioRad, Madrid, Spain) to check integrity. A total of 12 RNA-seq experiments were performed, comprising two replicates for each ripening stage and melatonin treatment. Library preparation and sequencing were performed by the High-Throughput Sequencing Unit (University of Malaga, Malaga, Spain) on a NextSeq550 platform (Illumina, Madrid, Spain), generating around 45 million paired-end reads (2 × 75 bp) per replicate. Raw reads were subjected to quality filtering before alignment to the *Capsicum annuum* L. reference transcriptome (NCBI UCD10Xc1.1) using Bowtie2 [82]. Transcript counts were quantified from the aligned reads using Samtools [83]. Differential Gene Expression (DGE) was rigorously determined using the DEgenes-Hunter pipeline [84], which provides an integrated framework of four distinct statistical algorithms: EdgeR, DESeq2, Limma, and NOISeq.

By cross-validating expression changes through these multiple robust methodologies, this approach ensures high-confidence normalization and minimizes the discovery of false positives in the differential expression profiles. For the ripening analysis of genes related to melatonin biosynthesis, the immature green pepper fruits (G) were established as the reference condition for comparison of gene expression levels.

KEGG pathway enrichment analysis was performed on the list of DEGs identified from the RNA-seq data using ShinyGO, which provides gene list analysis and graphical visualization of enriched pathways [85]. Gene annotation was conducted by mapping the DEGs to the Kyoto Encyclopedia of Genes and Genomes (KEGG) database, and enrichment analysis was carried out to identify significantly overrepresented pathways (https://www.genome.jp/kegg/mapper/convert_id.html, accessed on 27 February 2026). Statistical significance was determined using false discovery rate (FDR) correction (FDR < 0.05) together with a defined fold-change threshold (log_2_FC ≥ 1), and pathways with adjusted *p*-values below the selected threshold were considered significantly enriched.

### 2.8. Melatonin Detection and Quantification via the UPLC Method with Fluorescence Detection (FD)

Melatonin extraction from pepper fruits was performed using a method previously described [86,87,88], with some minor modifications. In brief, 9 mL of ethyl acetate was added to 0.3 g of pepper fruit in glass centrifuge tubes. After being placed in a rotating agitator for 15 h at 20 °C in darkness, the samples were centrifuged for 10 min at 5000× *g* using a Sorvall RC 5B Plus centrifuge (DuPont, Wilmington, DE, USA). After centrifugation, the ethyl acetate was evaporated with N_2_ gas in the dark. Once all the acetate has evaporated, the remaining residue is suspended in 1 mL of acetonitrile, and an agitator vortex was used to ensure homogeneity before filtration through a polytetrafluoroethylene (PTFE) filter with a 0.22 μm pore size.

Each sample was injected at least three times. Finally, the melatonin content of the samples was analyzed using ultra-high performance liquid chromatography (UPLC, H-Class, Waters, Mildford, MA, USA), coupled to a fluorescence detector (UPLC-FD). An ACQUITY UPLC HSS T3 1.8 µm 2.1 × 100 mm column with a VANGUARD 2.1 × 5 mm precolumn (Waters) was used, employing a 15 min gradient elution method. The excitation and emission wavelengths were 280 nm and 340 nm, respectively. The mobile phase (Fm) composition consisted of H_2_O + 0.1% formic acid (Fm A) and methanol (Fm B) at a flow rate of 0.3 mL/min, with an initial working ratio of 95:5 (A:B). Elution was performed using a gradient method, progressing from the initial conditions to 5:95 over 10 min. Under these conditions, melatonin eluted in 4.639 min. Five microliters of each sample were injected, with the column temperature at 45 °C. The method was validated with parameters, including the limit of detection (LOD), the limit of quantitation (LOQ), linearity, precision, and accuracy (Table 1).

### 2.9. Electrochemical Detection of Total Antioxidant Capacity (TAC) in Pepper Fruit Treated with Melatonin

The total antioxidant capacity (TAC) of the pepper fruits during ripening and treated with melatonin was quantified using the e-BQC portable laboratory device (Bioquochem S.L., BQC Redox Technologies, Asturias, Spain) in accordance with the manufacturer’s protocol using ascorbate as reference [91]. The method is based on the electrochemical determination of the sample’s redox potential, expressed as charge per period in microcoulombs (μC), thereby providing a direct measurement of TAC as the total charge (μC) corresponding to the electrons capable of reducing or neutralizing free radicals. The statistical analysis of the obtained TAC data was conducted using one-way analysis of variance (ANOVA), followed by appropriate pairwise comparisons, employing Statgraphics Centurion software 19. Differences were deemed statistically significant at *p* < 0.05.

### 2.10. Statistical Analyses

Different statistical approaches were employed based on the specific data sets analyzed. The statistical analysis of the obtained TAC data was conducted using one-way analysis of variance (ANOVA), followed by appropriate pairwise comparisons, employing Statgraphics Centurion software. Differences were deemed statistically significant at *p* < 0.05. For the quantification of the red-colored area (expressed as total pixels), statistical analyses were performed using R 4.5.2 software. The assumptions of normality and homogeneity of variances were previously verified using the Shapiro-Wilk and Levene’s tests, respectively. To determine the effect of the melatonin treatments and time on the reddening process, a two-way analysis of variance (ANOVA) was conducted. For multiple comparisons among treatments within each specific day, Tukey’s Honestly Significant Difference (HSD) post-hoc test was applied. Differences were considered statistically significant at *p* < 0.05. Results are expressed as the mean ± standard error (SE) of the technical replicates. Data visualization, including bar charts with statistical significance letters, was generated using the ggplot2 package 4.0.2.

## 3. Results

### 3.1. Identification of the Genes Encoding the Enzymes Involved in Melatonin Biosynthesis in Pepper and Their Subcellular Location

In a previous study, we identified five genes that encode the enzyme tryptophan decarboxylase (TDC) [40]. However, to date, the genes responsible for encoding the other enzymes involved in melatonin biosynthesis in sweet peppers have not been fully identified. Therefore, Table 2 presents a summary of the additional identified genes encoding enzymes involved in the melatonin biosynthetic pathway, with those expressed in fruits highlighted in blue.

### 3.2. Analysis of Gene Structure and Regulatory Elements of the Genes Encoding Enzymes Participating in Melatonin Biosynthesis

**Figure 2:**
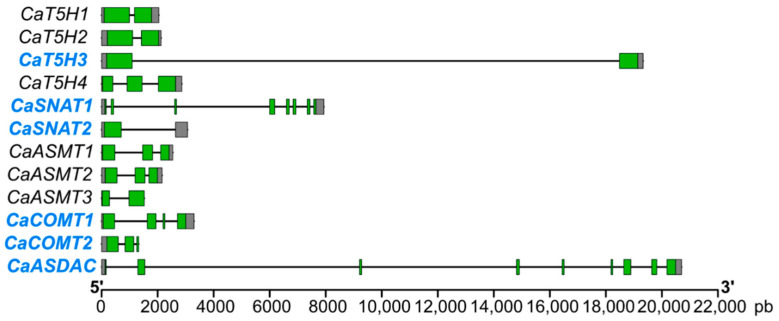
Genomic organization of the twelve genes identified in pepper plants that are involved in melatonin biosynthesis (*T5H*, *tryptamine 5-hydroxylase*; *SNAT*, *serotonin N-acetyltransferase*; *ASMT*, *N-acetylserotonin O-methyltransferase*; *COMT*, *caffeic acid O-methyltransferase*; and *ASDAC*, *N-acetylserotonin deacetylase*). The gene structure is illustrated with exons represented by green boxes, introns by black lines, and untranslated regions by gray boxes. Exon–intron regions are drawn to scale. Blue color indicates the six genes expressed in pepper fruits.

The genomic organization of the *C. annuum* genes encoding enzymes for melatonin biosynthesis, as illustrated in Figure 2, displays significant structural variability, ranging from compact genes with only two exons (e.g., *CaASMT1*-3, and *CaCOMT1*-2) to very large and complex genes spanning over 20,000 pb (*CaASDAC* and *CaT5H3*). This contrast highlights that while the majority of genes encoding enzymes, particularly those in the final methylation steps (*CaASMT* and *CaCOMT* families), feature a conserved, simple two-exon structure, genes involved in earlier steps, specifically the key regulatory gene, *CaT5H3*, along with *CaASDAC*, are substantially larger due to either multiple exons or long introns, suggesting potential differences in transcriptional regulation or the possibility of alternative splicing leading to functional diversity. Focusing on the six genes expressed in the *C. annuum* fruit (*CaT5H3*, *CaSNAT1*, *CaSNAT2*, *CaCOMT1*, *CaCOMT2*, and *CaASDAC*), which constitute the potential melatonin biosynthetic pathway in this organ, the heterogeneity remains evident: the majority, especially those involved in later steps, maintain the simple, compact two-exon structure, which implies a genetic design for robust and consistent enzyme production crucial for antioxidant or signaling activity in the fruit; conversely, the two essential regulatory genes, *CaT5H3* and *CaASDAC*, possess the large and complex structures that strongly suggest complex transcriptional control and alternative splicing are key mechanisms for fine-tuning the rate and timing of melatonin production in the developing pepper fruit.

**Figure 3:**
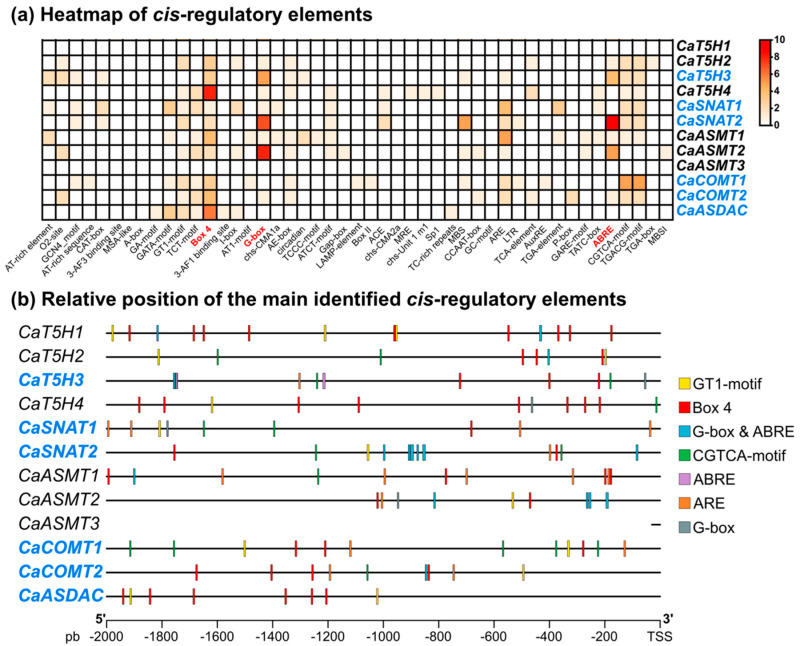
(**a**) Heatmap of *cis*-regulatory elements in the 2000 bp region upstream of the transcription start site of the identified genes involved in the biosynthesis of melatonin in pepper, including *CaT5H*s, *CaSNAT*s, *CaASMT*s, *CaCOMT*s, and *CaASDAC*. In blue color appear those genes which are expressed in fruits. The *cis*-regulatory elements are categorized based on their functional roles, including regulation, light response, stress response, and phytohormones. The *cis*-regulatory elements were identified using the PlantCARE database. (**b**) Relative position of the main identified *cis*-regulatory elements in the promoter regions of melatonin-related genes in pepper. The diagram displays the distribution of various regulatory motifs within a 2000 bp region upstream of the transcription start site (TSS) for genes involved in melatonin biosynthesis. Specific *cis*-elements are represented by colored bars, including the GT1-motif (yellow), Box 4 (red), G-box & ABRE (cyan), CGTCA-motif (green), ABRE (purple), ARE (orange), and G-box (gray). These motifs indicate potential regulation by light (Box 4, GT1-motif), abscisic acid (ABRE), methyl jasmonate (CGTCA-motif), and anaerobic induction (ARE), highlighting a complex regulatory network governing melatonin levels in response to diverse environmental and hormonal signals.

The regulatory elements involved in controlling gene expression were also analyzed to assess their putative regulatory potential. *Cis*-regulatory elements located within the 2000 bp upstream of the transcription start site of each gene were identified (Figure 3a). Among all elements examined, Box 4, G-Box, and ABRE exhibited the highest enrichment and are associated with light responsiveness and phytohormone signaling, indicating that the expression of these biosynthetic genes is strongly influenced by environmental light conditions and hormonal regulation. Box 4, a light-responsive element, shows significant presence across nearly all analyzed genes, with particularly strong enrichment in *CaT5H1*, *CaT5H4*, and *CaASDAC*. This suggests that these genes may represent primary targets of light-mediated regulation, potentially contributing to circadian-dependent synthesis. The G-Box, another well-characterized light-responsive element that interacts with bZIP transcription factors, is especially enriched in *CaT5H3*, *CaSNAT2*, and *CaASMT2*, indicating that later steps of the pathway are particularly sensitive to light-induced activation. The abscisic acid-responsive element (ABRE) exhibits pronounced enrichment, most notably in *CaSNAT2*, suggesting a strong regulatory responsiveness to abscisic acid (ABA) and osmotic stress. Moderate-to-high enrichment of ABRE in *CaT5H3* and *CaASMT2* further supports a potential role for these genes in stress-adaptive responses. Collectively, the abundance and distribution of these cis-elements highlight their putative regulatory potential in modulating gene expression in response to environmental and hormonal cues. Furthermore, Figure 3b presents the relative positions of the principal cis-regulatory elements identified within a 2000 bp region upstream of the transcription start site (TSS) in the promoter regions of melatonin-related genes in pepper. In the case of *CaASMT3*, no cis-regulatory elements were identified, likely due to the very limited upstream sequence available (only 30 nucleotides). However, this absence should be interpreted cautiously, as such elements may reside outside the analyzed region, including further upstream, downstream, within introns, or in untranslated regions (UTRs).

### 3.3. Phylogenetic Trees of CaT5H, CaSNAT, CaASMT, CaCOMT, and CaASDAC Isozymes

Phylogenetic analyses of the different isozymes of pepper involved in the biosynthesis of melatonin, including *CaT5H*, *CaSNAT*, *CaASMT*, *CaCOMT*, and *CaASDAC*, were conducted alongside other orthologous proteins found in different plant species. These findings are illustrated in Figures S1–S5, and the corresponding identifiers are listed in Supplementary Tables S1–S5.

The phylogenetic tree of T5Hs (Supplementary Figure S1) across species like *C. annuum* and *A. thaliana* illustrates a complex evolutionary history of gene duplication and functional specialization, where the enzymes are categorized into distinct clades (Ia, Ib, IIa, IIb, and III) based on their sequence homology and divergence. While Clade I often represents ancestral sequences responsible for basal serotonin synthesis, Clades II and III frequently show evidence of gene expansion through tandem duplication, particularly in the pepper genome, leading to tissue-specific roles in the roots or leaves and specialized responses to abiotic stressors. The separation of Arabidopsis homologs from the Solanaceae clusters further underscores the deep evolutionary split between these plant families, suggesting that while the core biochemical pathway for serotonin production is conserved, the regulatory mechanisms and environmental adaptations provided by these various T5H isoforms have evolved independently to meet the specific ecological needs of each species.

The enzymes CaSNAT1 and CaSNAT2 are phylogenetically distinct from one another, yet both are closely related to the SNAT proteins in other plant species within the Solanaceae family, such as tomato (*Solanum lycopersicum*), tobacco (*Nicotiana tabacum*), and potato (*Solanum tuberosum*) (Supplementary Figure S2). In contrast, the OsSNATs proteins are more closely aligned with those of monocotyledonous plants, including maize (*Zea mays*), Bermuda grass (*Brachypodium distachyon*), barley (*Hordeum vulgare*), and wheat (*Triticum aestivum*). Further examination of the homology between tomato SNATs (SlSNATs) and those found in peppers reveals a 91.6% similarity between SlSNAT1 and CaSNAT1, along with a 79.5% similarity between SlSNAT2 and *CaSNAT2*. This suggests that there are two significantly different proteins within this plant family, both exhibiting serotonin *N-acetyltransferase* activity, given that the homology between SlSNATs is 33%. Additionally, it appears that most plant species possess two proteins with SNAT activity.

In Supplementary Figure S3, CaASMT1, CaASMT2, and CaASMT3 proteins cluster within Cluster Ia, reflecting a close evolutionary relationship with dicots such as tomato and Arabidopsis. Their tight grouping suggests they arose from Capsicum-specific gene duplications. These enzymes are essential for melatonin biosynthesis, aiding potential adaptation to stresses like drought [92] and temperature fluctuations, though they are not expressed in pepper fruit. Supplementary Figure S4 shows CaCOMTs largely within dicot-specific clades (Cluster Ia), closely related to tomato and potato. The *CaCOMT* family has expanded in pepper, forming clusters indicative of functional diversification beyond lignin biosynthesis. Notably, CaCOMTs often overtake ASMTs in converting *N-*acetylserotonin, highlighting their role in melatonin production, stress tolerance, and fruit ripening. Supplementary Figure S5 confirms CaASMT proteins’ placement in Cluster Ia, closely related to tomato (SlASMT) and Arabidopsis (AtASMT), and distinct from monocots like rice or wheat, emphasizing their Solanaceae-specific evolution.

### 3.4. Melatonin Quantification During Fruit Ripening by UPLC-FD

**Figure 4:**
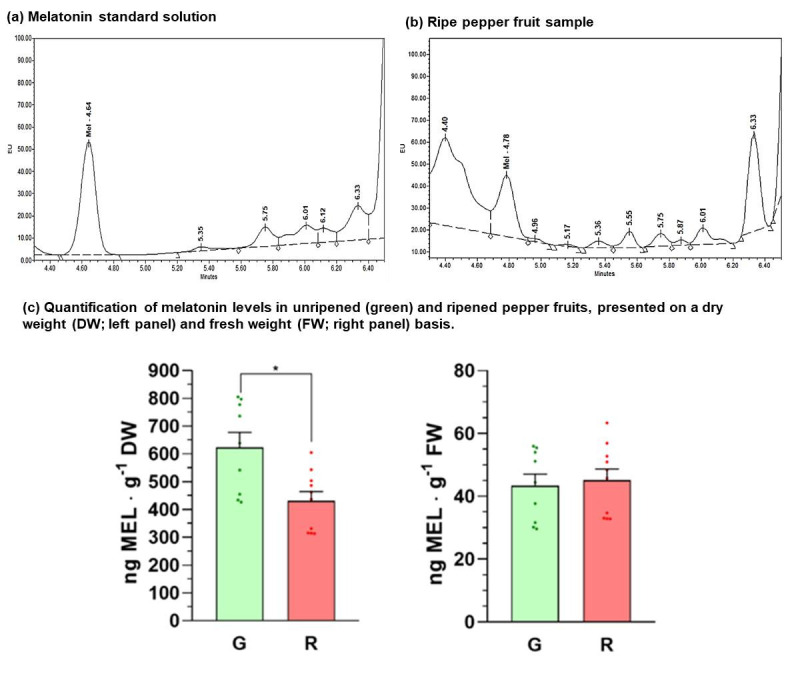
Melatonin quantification using the UPLC method with fluorescence detection in green and red pepper fruits. (**a**) Representative chromatogram of melatonin standard solution (70 ng·mL^−1^). (**b**) Representative chromatogram of melatonin detection in a ripe pepper fruit sample. The mobile phase (Fm) consisted of H_2_O with 0.1% formic acid (Fm A) and methanol (Fm B), delivered at a flow rate of 0.3 mL/min, with an initial ratio of 95:5 (A:B). EU, Emission Units. (**c**) Quantification of melatonin levels in unripe (green) and ripened pepper fruits (red), presented on a dry weight (DW; left panel) and fresh weight (FW; right panel) basis. The data are the result of a minimum of eight different biological samples with three replicates of each. An asterisk indicates that the differences were significant at *p* < 0.05 in Student’s *t*-test.

Figure 4a,b present representative chromatograms for the detection of melatonin in the standard (retention time (RT) = 4.639 min) and the ripened pepper fruit sample (RT = 4.783 min), respectively. The observed 0.144 min deviation in retention time is likely indicative of matrix effects (e.g., altered column equilibrium) induced by the complex composition of the fruit sample. Figure 4c shows the quantification of melatonin in unripe (green) and ripened sweet pepper fruits, which revealed concentrations of 623 ng melatonin·g^−1^ dry weight (DW) in green fruits and 431 ng melatonin·g^−1^ DW in red fruits, indicating that the melatonin content is reduced 31%. To express the melatonin content during fruit ripening, it was decided to do so by dry weight of the sample, given the high water content of pepper fruits, which is 93% in green fruits and 89.5% in red fruits. When expressed in fresh weight, no significant differences are observed due to the loss of water content that occurs during ripening.

### 3.5. RNAseq Analysis of Pepper Fruits During Pepper Fruit Ripening and After Treatment with Melatonin (0, 20, 50, and 100 µM)

**Figure 5:**
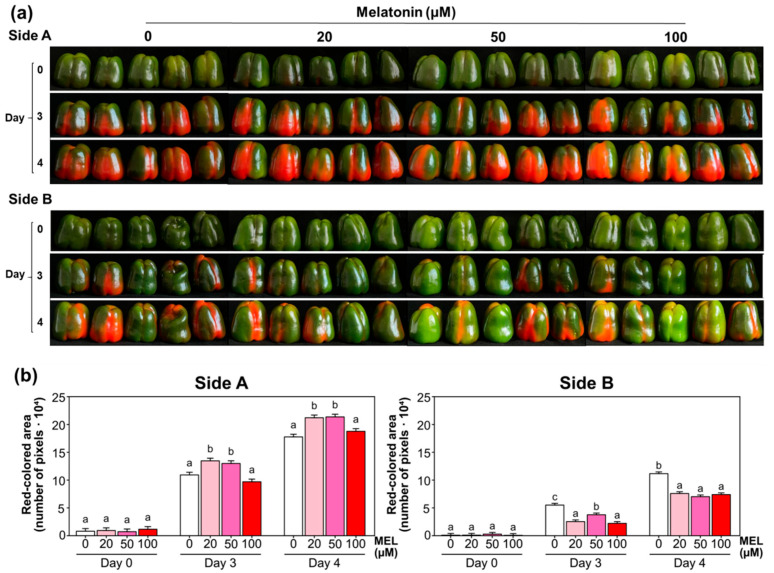
Phenotype of pepper (*C. annuum* cv. Masami) fruits treated with exogenous melatonin at concentrations of 20, 50, and 100 μM. Treatments were applied by spraying onto fruits at the onset of veraison, which were then kept at room temperature and photographed on the day of treatment and after 3 and 4 days. (**a**) Side A and B, showing the higher and lower degrees of ripeness, respectively. (**b**) Quantification of the area corresponding to the red color, expressed as a pixel count. Data represent the mean of five independent biological replicates per treatment. Different lowercase letters denote statistically significant differences among treated and untreated fruits within the same day, according to Tukey’s HSD test (*p* < 0.05).

Figure 5a shows the phenotype of pepper (*C. annuum* cv. Masami) fruits at the breaking point treated with exogenous melatonin (20, 50, and 100 μM) after 3 and 4 days. The progression of pepper ripening was evaluated by quantifying the total red-colored area. Images of the pepper pools (Side A and Side B) were captured at different time points (Day 0, Day 3, and Day 4) across all experimental groups (control and three melatonin concentrations). To account for measurement precision, the total number of red pixels in each image was quantified in triplicate, serving as technical replicates for each treatment pool at each specific time point. Figure 5b display the quantification of the red-colored area (expressed as total pixels) for Side A and Side B at days 0, 3, and 4. On side B, a noticeable decrease in red color content was observed on the third and fourth days of treatment with melatonin. This suggests that melatonin may be delaying the ripening of the peppers.

**Figure 6:**
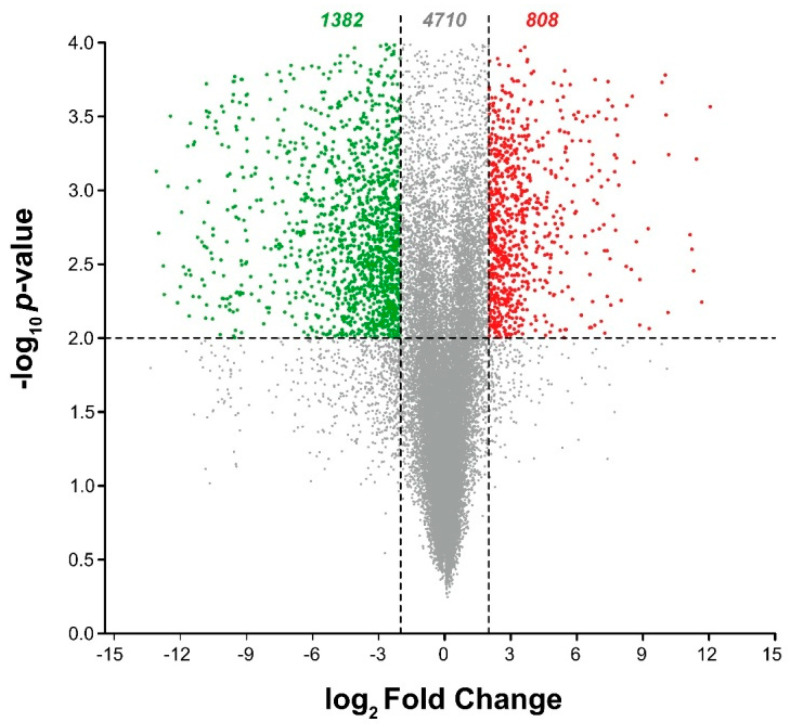
Volcano Plot of Differentially Expressed Genes (DEGs) in sweet pepper fruit cv. Masami during ripening. The plot visualizes the results of the differential expression analysis from RNA-sequencing data comparing ripe versus unripe pepper fruit. The *X*-axis represents the log_2_ Fold Change, indicating the magnitude and direction of gene expression change. The *Y*-axis represents the statistical significance as the negative base-10 logarithm of the *p*-value (−log_10_ *p*-value). The dashed lines indicate the thresholds for identifying significant DEGs: log_2_ FC ≥ 2 or log_2_ FC *≤* −2 and −log_10_ *p*-value ≥ 2.0 (corresponding to *p* ≤ 0.01). Red dots represent genes significantly upregulated in ripe fruit (log_2_ Fold Change ≥ 2). Green dots represent genes significantly downregulated in ripe fruit (log_2_ FC ≤ −2). Gray dots are genes that are not considered significantly differentially expressed.

Considering that fruit ripening is a highly regulated and complex process, RNA-seq analysis was performed to obtain a comprehensive molecular framework to dissect both the intrinsic ripening program of pepper fruits and the regulatory effects of exogenous melatonin. For this purpose, green and red fruits, as well as fruits treated with melatonin, after 4 days, were selected for subsequent RNA-seq analysis. Figure 6 illustrates the volcano plot analysis that summarizes the differential gene expression analysis from RNA-seq data comparing unripe (green) and ripe (red) pepper fruit. The *X*-axis plots the log_2_ Fold Change (FC), indicating the magnitude of expression difference, while the *Y*-axis plots the statistical significance as the –log_10_ *p*-value. Genes are considered significantly differentially expressed (DEGs) if they meet the thresholds set by the dashed lines: a log_2_ FC ≥ 2 (or Fold Change ≤ −2) and a –log_10_ *p*-value ≥ 2.0 (*p* ≤ 0.01). The red dots in the upper right quadrant represent 808 genes significantly upregulated during ripening, while the green dots in the upper left quadrant represent 1382 genes preferentially expressed in the unripe stage. The numerous gray dots represent 4710 genes that did not meet one or both significance criteria. The plot demonstrates a high degree of transcriptional reprogramming, as evidenced by the large number of genes, particularly those shown in red, that are strongly and significantly altered in expression as the pepper fruit transitions from the unripe to the ripe stage. Additionally, Supplementary Table S6 provides a summary of the raw and trimmed read counts obtained from sequencing the 12 cDNA libraries.

**Figure 7:**
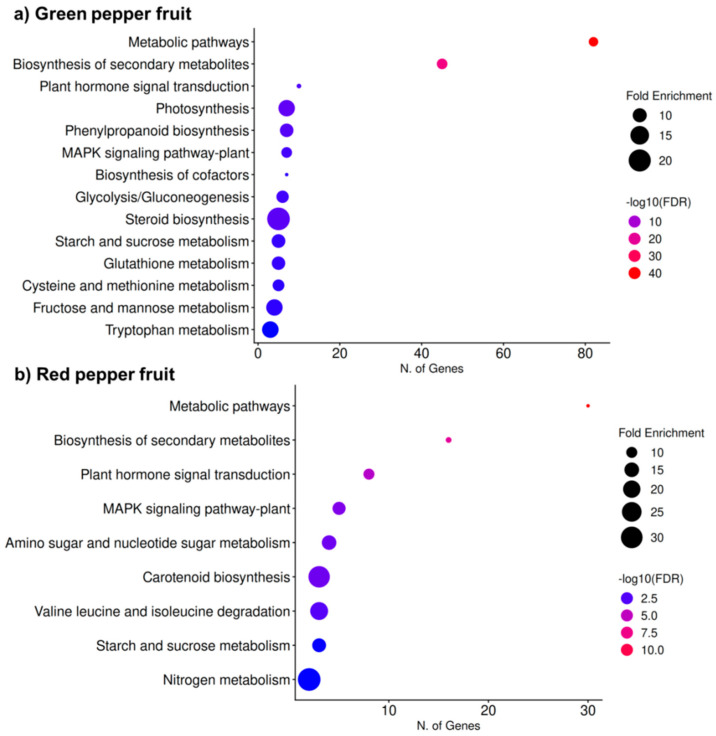
KEGG pathway enrichment analysis of differentially expressed genes identified by means of RNA-seq in pepper fruits. (**a**) Green pepper fruit. (**b**) Red pepper fruit. The dot plots show the significantly enriched KEGG pathways in each developmental stage. The *X*-axis indicates the number of genes associated with each pathway, and the *Y*-axis lists the corresponding KEGG categories. Dot size represents fold enrichment, while color indicates statistical significance expressed as −log10 (FDR). Larger dots and warmer colors (red) correspond to higher enrichment and greater statistical significance, respectively. FDR, false discovery rate.

The KEGG enrichment analysis revealed distinct metabolic signatures between green and red pepper fruits (Figure 7). In green fruits (Figure 7a), the most significantly enriched pathways were mainly associated with primary metabolism and stress-related processes, including metabolic pathways, biosynthesis of secondary metabolites, plant hormone signal transduction, photosynthesis, phenylpropanoid biosynthesis, MAPK signaling, and carbohydrate metabolism (starch and sucrose, glycolysis/gluconeogenesis, fructose and mannose metabolism). Glutathione metabolism and cysteine and methionine metabolism were also enriched, suggesting active redox regulation at this stage. In contrast, red fruits (Figure 7b) showed enrichment in pathways more closely related to ripening and specialized metabolism, such as carotenoid biosynthesis, amino sugar and nucleotide sugar metabolism, nitrogen metabolism, and branched-chain amino acid degradation, along with plant hormone signal transduction and MAPK signaling. Overall, while green fruits exhibit stronger enrichment in photosynthesis and general metabolic processes, red fruits display a metabolic shift toward pathways associated with fruit maturation and pigment accumulation.

**Figure 8:**
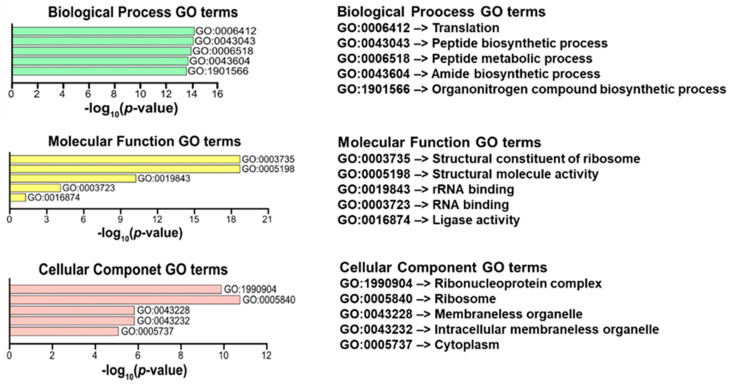
Clustering by the top 5 most relevant Gene Ontology (GO) terms of the 31,373 genes significantly regulated by MEL treatment in sweet pepper fruits cv. Masami.

Figure 8 presents the results of a Gene Ontology (GO) enrichment analysis of the 31,373 genes significantly regulated by MEL treatment in sweet pepper fruits, highlighting the five most relevant terms for the input gene set across the three main GO categories: Biological Process, Molecular Function, and Cellular Component. The *X*-axis for all panels represents the statistical significance of the enrichment, calculated as the negative decadic logarithm of the *p*-value (−log_10_ *p*-value). The length of each bar is proportional to the significance, where a longer bar indicates a more significantly enriched term (a smaller *p*-value). Panels and Key Findings: Biological Process (Top): The most significantly enriched term is GO:0006412 (Translation), highlighting that the primary collective function of the target genes is protein synthesis. Molecular Function (Middle): The most significant term is GO:0003735 (Structural constituent of ribosome), indicating that the gene products physically make up the machinery for translation. Cellular Component (Bottom): The most significant term is GO:1990904 (Ribonucleoprotein complex), which includes the ribosome structure itself, confirming the cellular location of the gene functions. Therefore, the enrichment analysis strongly suggests that the input genes are central to ribosome function and the process of translation.

### 3.6. Expression of Melatonin Biosynthetic Genes in Fruits at Ripening and After Treatment with Melatonin (0, 20, 50, and 100 µM)

**Figure 9:**
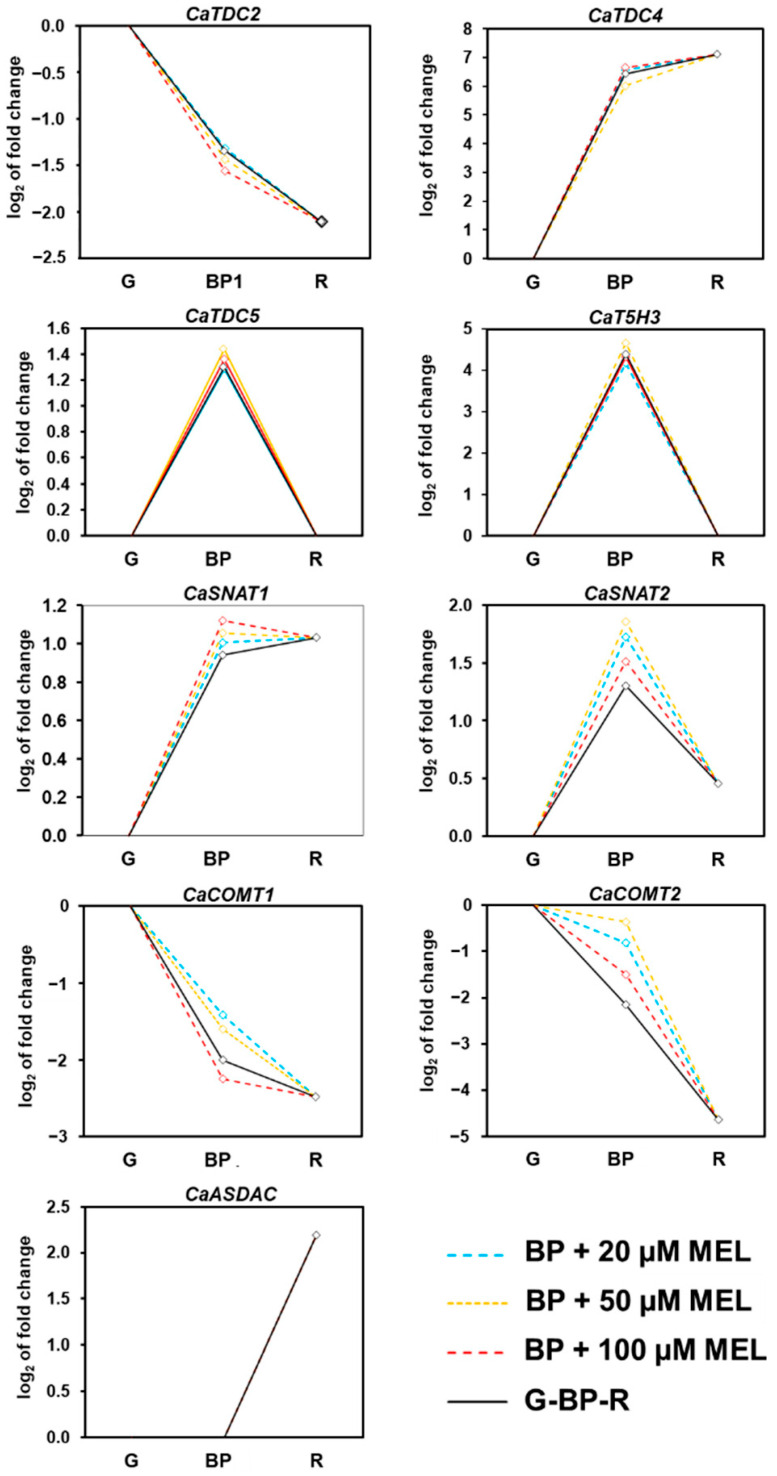
Time-course RNA-seq analysis of the nine identified melatonin biosynthetic genes [*tryptophan decarboxylase* (*TDC*), *tryptamine 5-hydroxylase* (*T5H*), *serotonin N-acetyltransferase* (SNAT), *caffeic acid O-methyltransferase* (*COMT*), and *N-acetylserotonin deacetylase* (*ASDAC*)] during pepper fruit and after exogenous melatonin (MEL) treatment (20, 50, and 100 µM). Samples of sweet pepper fruits cv. Masami at different ripening stages correspond to immature green (G), breaking point (BP), and ripe red. Statistically significant changes in expression levels (*p* < 0.05) compared to green fruit are indicated with diamonds.

To determine whether exogenous melatonin modulates the expression of genes involved in its own biosynthesis, RNAseq analysis was performed on green and red pepper fruits, as well as on fruits at the breaking point treated with 20, 50, or 100 µM melatonin. The resulting expression profiles illustrate the transcriptional dynamics of melatonin biosynthetic genes during sweet pepper fruit ripening and their responses to exogenous melatonin at different concentrations, as presented in Figure 9. The transcriptional patterns of these genes exhibited distinct behaviors throughout fruit ripening. Specifically, *CaTDC2* was downregulated, whereas *CaTDC4* showed a marked increase in expression. In contrast, *CaTDC5* and *CaT5H3* were not significantly affected. Both *CaSNAT1* and *CaSNAT2* were upregulated, with a higher expression level observed for *CaSNAT1*. Conversely, *CaCOMT1* and *CaCOMT2* were significantly downregulated, while *CaASDAC* displayed upregulation during ripening. Under treatments with exogenous melatonin at varying concentrations, the expression of these genes was also differentially modulated. The most pronounced effects were observed for *CaSNAT1* and *CaSNAT2*, for which all melatonin concentrations induced a significant upregulation. A similar response was detected for *CaCOMT2*. Interestingly, *CaCOMT1* was upregulated under 20 and 50 µM melatonin treatments but downregulated at 100 µM.

**Figure 10:**
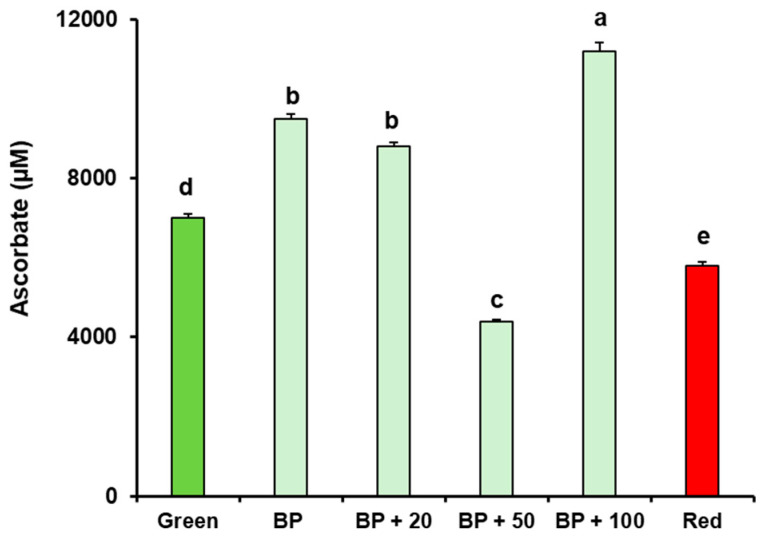
Total antioxidant capacity (TAC) expressed in equivalent of μM ascorbate during pepper fruit and after exogenous melatonin (MEL) treatment with 20, 50, and 100 µM. Samples of sweet pepper fruits cv. Masami at different ripening stages correspond to immature green, breaking point (BP), and ripe red. Data are presented as means ± SEM from at least three biological samples, each with a minimum of three repetitions. Different letters indicate statistically significant differences among values at *p* < 0.05, as determined via ANOVA.

To evaluate the potential effect of the exogenous melatonin, the total antioxidant capacity (TAC) expressed in equivalent of μM ascorbate in pepper fruit at the breaking point stage was evaluated and compared with green (immature) and red (mature) fruits. Thus, Figure 10 shows that TAC decreases in red peppers compared with green peppers. In BP fruits treated with melatonin, TAC decreases at 50 µM but increases at 100 µM, indicating a dose-dependent response. Therefore, for future studies, the 100 µM concentration would be selected.

**Figure 11:**
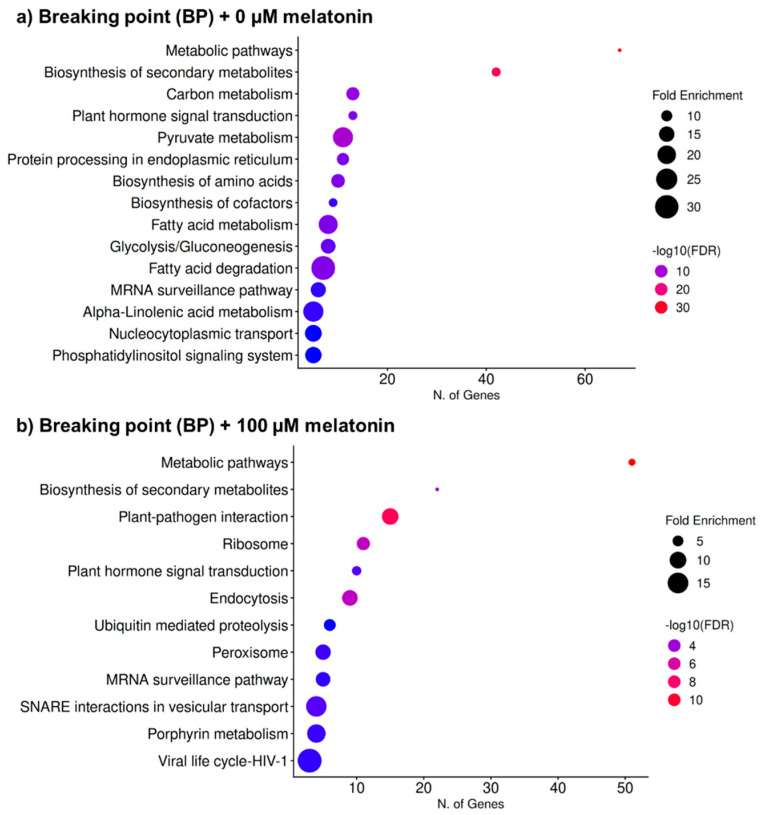
KEGG pathway enrichment analysis of differentially expressed genes identified by means of RNA-seq in pepper fruits. (**a**) Fruit at breaking point (BP) + 0 µM melatonin. (**b**) Fruit at breaking point (BP) + 100 µM melatonin-treated red pepper fruit. The dot plots show the significantly enriched KEGG pathways in each developmental stage. The *X*-axis indicates the number of genes associated with each pathway, and the *Y*-axis lists the corresponding KEGG categories. Dot size represents fold enrichment, while color indicates statistical significance expressed as −log10 (FDR). Larger dots and warmer colors (red) correspond to higher enrichment and greater statistical significance, respectively. FDR, false discovery rate.

To explore the potential molecular mechanisms underlying the delay in ripening induced by the exogenous application of melatonin, a KEGG pathway analysis was conducted on pepper fruits at the breaking point (BP). Both untreated (0 μM) and 100 μM melatonin-treated samples were analyzed, with particular emphasis on genes involved in plant hormone signal transduction (Figure 11). In both conditions, “metabolic pathways” and “biosynthesis of secondary metabolites” are the most enriched categories, with the highest number of genes and strongest statistical significance. As expected, this indicates that fruit ripening is largely driven by extensive metabolic reprogramming, which remains a central feature even after melatonin treatment. In untreated fruits (Figure 11a), enrichment is more strongly associated with core metabolic processes, including carbon metabolism, glycolysis/gluconeogenesis, fatty acid metabolism, and biosynthesis of amino acids and cofactors. Additionally, pathways such as protein processing in the endoplasmic reticulum and mRNA surveillance suggest active protein turnover and quality control during ripening. The prominence of α-linolenic acid metabolism also points to lipid remodeling and potential oxylipin signaling. In contrast, melatonin-treated fruits (Figure 11b) show a shift toward pathways related to cellular regulation and stress responses. Notably, there is enrichment in plant–pathogen interaction, endocytosis, ubiquitin-mediated proteolysis, and SNARE (Soluble-ethylmaleimide-sensitive factor Attachment protein Receptor)-mediated vesicular transport, indicating enhanced signaling, protein trafficking, and turnover. The enrichment of the peroxisome pathway is particularly relevant, as it supports a role for melatonin in modulating redox metabolism and reactive oxygen and nitrogen species (ROS and RNS) homeostasis. The presence of porphyrin metabolism may also reflect adjustments in tetrapyrrole-related processes linked to oxidative status. Interestingly, plant hormone signal transduction appears in both conditions, but its relative contribution differs, suggesting that melatonin may interact with or modulate hormonal networks during ripening. Thus, these analyses suggest that while basal ripening is dominated by metabolic and biosynthetic pathways, melatonin treatment redirects gene expression toward enhanced stress adaptation, redox regulation, and intracellular trafficking, consistent with its known role as a signaling molecule with antioxidant properties.

**Figure 12:**
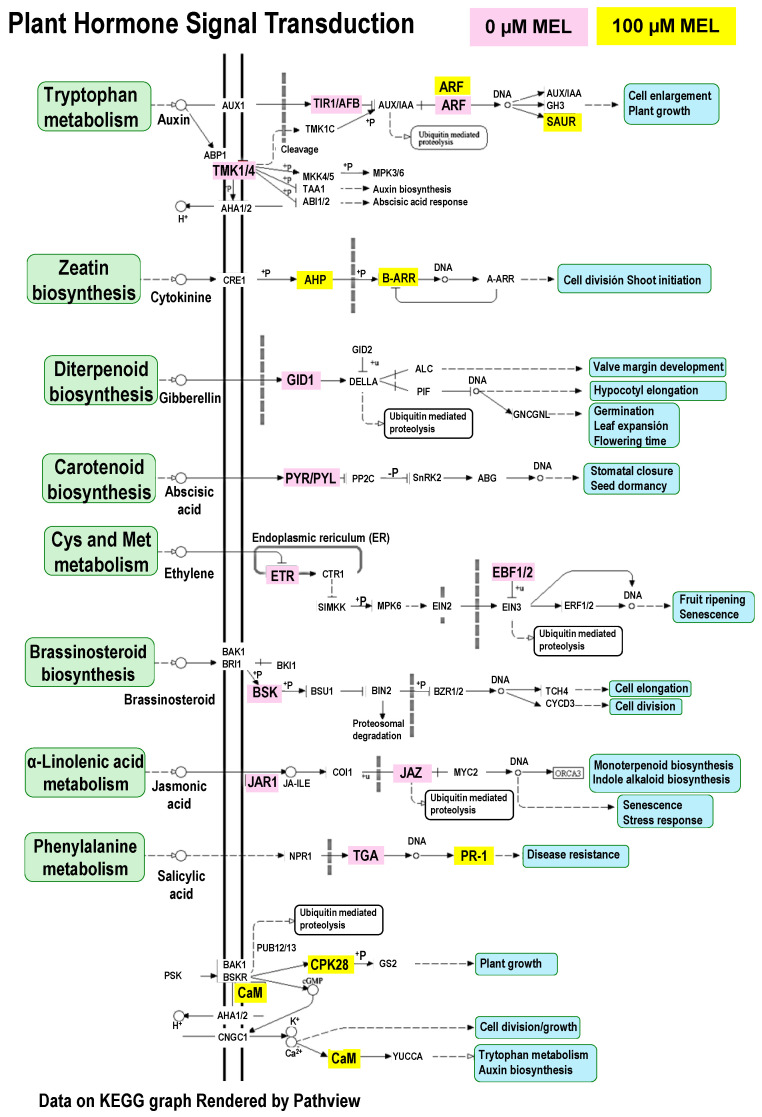
Comparison between untreated and 100 µM melatonin-treated pepper fruits at the breaker stage (BP), focusing on genes involved in Plant Hormone Signal Transduction Pathways identified through KEGG analysis. Differentially expressed genes in pepper fruits at the breaking stage (PB) are highlighted in purple for untreated samples and in yellow for those treated with 100 µM melatonin. Abbreviations: *TIR1/AFB*, *TRANSPORT INHIBITOR RESPONSE1/AUXIN SIGNALING F-BOX*; *ARF*, *AUXIN RESPONSE FACTOR*; *SAUR*, *Small Auxin Up-Regulated RNA*; *AHP*, *Arabidopsis Histidine Phosphotransfer Proteins*; *B-ARR*, *Type-B Arabidopsis Response Regulators*; *GID1*, *GIBBERELLIN-INSENSITIVE DWARF1*; *PYR/PYL*, *PYRABACTIN RESISTANCE 1*/*PYR1-LIKE* regulatory components of ABA receptor; *ETR*, *Ethylene Response*; ERF1/2, *Ethylene Response Factor 1/2*; *BSK*, *BRASSINOSTEROID-SIGNALING KINASE*; *JAR1*, *JASMONATE RESISTANT 1*; *JAZ*, *JASMONATE ZIM-DOMAIN*; *TGA*, *TGACG-motif-binding factor*; *PR-1*, *Pathogenesis-Related protein 1*; *CML*, *Calmodulin-like protein*; and, *CPK*, *Calcium-dependent protein kinase*.

Considering the previous results, the specific analysis of the gene involved in plant hormone signal transduction identified in KEGG pathways is shown in Figure 12. Thus, melatonin (100 µM) treatment markedly altered plant hormone signal transduction in pepper fruits compared to untreated controls. In the auxin pathway, untreated fruits exhibited activation of upstream signaling components such as *TIR1/AFB* (*TRANSPORT INHIBITOR RESPONSE1/AUXIN SIGNALING F-BOX*) and *ARF* (*AUXIN RESPONSE FACTOR*), whereas melatonin-treated fruits showed a shift toward downstream response genes, including *SAUR* (*Small Auxin Up-Regulated RNA*). Cytokinin signaling was enhanced following melatonin application, as evidenced by the upregulation of *AHP* (*Arabidopsis Histidine Phosphotransfer Proteins*) and *B-ARR* (*Type-B Arabidopsis Response Regulators*). Conversely, several hormone pathways associated with stress and ripening were downregulated by melatonin. Key genes involved in gibberellin (*GID1*, *GIBBERELLIN-INSENSITIVE DWARF1*), abscisic acid (*PYR/PYL*, *PYRABACTIN RESISTANCE 1*/*PYR1-LIKE* regulatory components of ABA receptor), ethylene (*ETR*, *Ethylene Response*; ERF1/2, *Ethylene Response Factor 1/2*), brassinosteroid (*BSK*, *BRASSINOSTEROID-SIGNALING KINASE*), and jasmonic acid (*JAR1*, *JASMONATE RESISTANT 1*; *JAZ*, *JASMONATE ZIM-DOMAIN*) signaling were prominently expressed in untreated fruits but showed reduced or no expression in treated fruits. Notably, salicylic acid (SA) signaling exhibited a shift from upstream regulation (*TGA*, *TGACG-motif-binding factor*) in controls to downstream defense activation (*PR-1*, *Pathogenesis-Related protein 1*) in melatonin-treated fruits. In addition, calcium signaling components, including *CML* (*Calmodulin-like protein*) and *CPK* (*Calcium-dependent protein kinase*), were specifically induced following melatonin treatment. Consequently, these results suggest that melatonin suppresses stress-related and ripening-associated hormonal pathways while promoting controlled growth signaling and efficient defense responses in pepper fruits.

## 4. Discussion

The importance of endogenous melatonin in higher plants, together with its biotechnological application, has gained increasing attention due to its antioxidant and signaling functions [5,7,11,26,93,94]. Exogenous melatonin delays ripening and extends shelf life in climacteric fruits such as banana [95,96], tomato [97], apple [98], peach [99], and cherimoya [100], as well as in non-climacteric fruits including strawberry [101] and sweet cherry [102]. In climacteric fruits, melatonin suppresses ethylene biosynthesis and signaling, partly through transcriptional regulation [98], modulates other ripening-related hormones such as abscisic acid [103], and enhances antioxidant capacity, metabolic stability, and sugar–organic acid balance, thereby reducing oxidative damage and quality loss. Overall, these findings establish melatonin as a multifunctional regulator of fruit ripening with strong potential for improving postharvest quality and storability.

As previously mentioned, the melatonin biosynthetic pathway has been mainly characterized in rice and *A. thaliana* [12,15,16,17,104], but remains incompletely understood in higher plants due to reversible steps and alternative routes. Consequently, increasing efforts have focused on other species, revealing limited knowledge of the genes encoding melatonin-biosynthetic enzymes and their organ-specific expression [105,106,107,108,109,110,111].

Pepper (*C. annuum* L.) is a major horticultural crop with extensive phenotypic diversity, comprising sweet and hot types [112]. Exogenous melatonin has been shown to enhance postharvest performance and stress tolerance in pepper fruits by alleviating chilling injury, improving antioxidant capacity, maintaining membrane stability, and delaying softening [24,113,114,115]. In seedlings, melatonin also improves cold tolerance by stimulating alkane synthesis in epicuticular wax [25]. Moreover, melatonin treatments influence fruit quality and regulate the expression of key melatonin-biosynthetic genes [116]. Despite these advances, the melatonin biosynthetic pathway in pepper fruits remains poorly defined. Here, we identified nine genes associated with melatonin biosynthesis in sweet California-type pepper fruits (cv. Masami) ([40], present study), all of which were expressed and transcriptionally regulated during ripening and in response to exogenous melatonin. This multiplicity suggests functional redundancy, potentially enhancing metabolic robustness under fluctuating environmental conditions. Notably, *ASMT* transcripts were undetectable in fruits, likely reflecting functional replacement by COMT enzymes in the final biosynthetic step. Consistently, Pan et al. (2019) [117] reported limited and highly tissue-specific *CaASMT* expression in pepper, supporting functional specialization within this gene family. Similar gene family complexity has been observed in quinoa and tomato [118,119].

Melatonin content declined by approximately 30% during pepper fruit ripening, consistent with earlier observations in diverse pepper cultivars [120], contrasting with increasing trends reported in tomato. This discrepancy highlights species- and genotype-dependent regulation, as well as the influence of tissue hydration, emphasizing the importance of dry-weight normalization for accurate metabolic interpretation.

KEGG analysis highlights carotenoid biosynthesis as a central hallmark of pepper fruit maturation, integrated with broader shifts in sugar and nitrogen metabolism. While red peppers typically show a strong enrichment in these pathways to facilitate pigment accumulation, melatonin treatment appears to disrupt this progression. Specifically, treated fruits exhibited a smaller red-colored area on days 3 and 4 (Figure 5, Side B), suggesting a delay in the ripening process. This implies that melatonin may act as a ripening inhibitor by modulating carotenoid biosynthesis and the underlying metabolic pathways that provide the necessary reducing power and carbon precursors. The particular analysis of the plant hormone signal transduction pathways identified through the KEGG pathway supports that melatonin treatment (100 µM) reconfigures pepper fruit physiology by shifting the hormonal balance from stress and ripening toward sustained growth and primed defense. By downregulating genes associated with abscisic acid, jasmonic acid, and gibberellin signaling, melatonin effectively delays maturity-related decay while simultaneously accelerating downstream responses in auxin and salicylic acid pathways. This metabolic reprogramming, supported by the induction of calcium signaling components like *CML* and *CPK*, suggests that melatonin acts as a major regulator that preserves fruit quality by prioritizing cellular maintenance and pathogen resistance over stress-induced senescence.

RNA-seq analysis revealed dynamic transcriptional regulation of melatonin-biosynthetic genes during ripening and following melatonin treatment, with distinct patterns among gene family members. The strong induction of *CaSNAT1* and *CaSNAT2*, together with dose-dependent modulation of *COMT* genes, indicates coordinated regulation and feedback control. These findings establish melatonin biosynthesis in pepper fruits as a tightly regulated process integrating developmental and signaling cues, reinforcing melatonin’s role in fruit ripening regulation.

## 5. Conclusions

**Figure 13:**
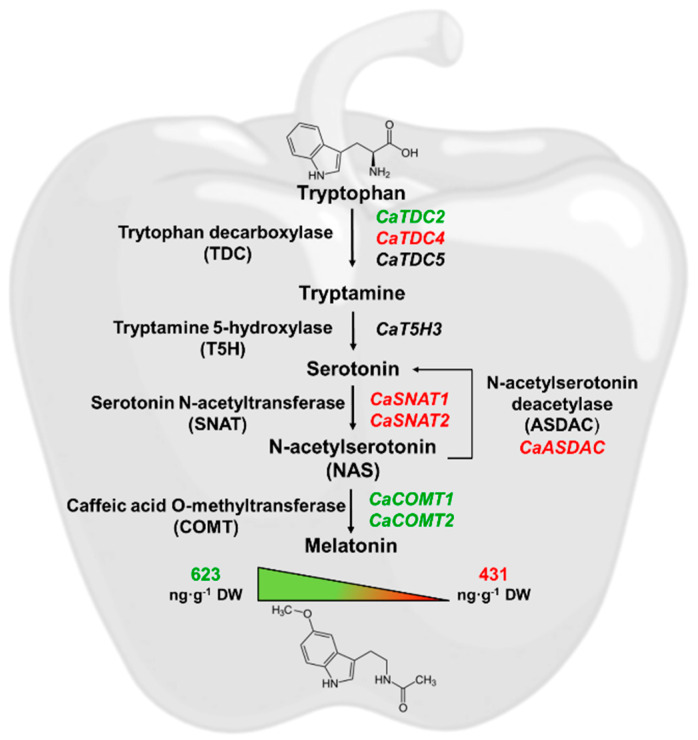
Working model illustrating the summary of genes involved in melatonin biosynthesis in pepper (*Capsicum annuum* L.) fruits. Genes predominantly expressed in immature fruits are shown in green, those predominantly expressed in ripe fruits are shown in red, and genes exhibiting no significant change are shown in black. T5H, tryptamine 5-hydroxylase. SNAT, serotonin N-acetyltransferase. ASMT, N-acetylserotonin O-methyltransferase. COMET, caffeic acid O-methyltransferase. ASDAC, N-acetylserotonin deacetylase.

Taken together, these findings have enabled the identification of the gene families encoding melatonin biosynthesis in sweet California-type pepper fruits. Although further studies are required to biochemically characterize the corresponding proteins, this genetic framework represents a crucial first step toward a comprehensive understanding of melatonin biosynthesis in pepper. Moreover, the expansion of these gene families likely reflects genome duplication events and provides functional redundancy, thereby enhancing metabolic robustness and stress resilience. The distinct expression patterns among duplicated genes indicate emerging specialization, allowing more precise regulation of melatonin production across tissues and developmental stages. Overall, this gene duplication strengthens the plant’s adaptive capacity. Of the 12 genes encoding proteins involved in melatonin biosynthesis in pepper, only 9 genes were detected in fruits. Moreover, the endogenous decrease in melatonin during fruit maturation suggests that exogenous treatment can decelerate ripening. This is evidenced by a significant reduction in red pigment accumulation, which serves as a reliable marker for carotenoid biosynthesis that characterizes pepper ripening. Figure 13 summarizes the identified melatonin-biosynthetic genes and their relative expression in unripe (green) and ripe (red) fruits.

## Figures and Tables

**Table 1:** Parameters of the linear regression (y = b + mx) obtained for the calibration curves of the standards of melatonin (1–100 μg·L^−1^), where b is the y intercept, m is the slope, r is the correlation coefficient and R^2^ is the squared correlation coefficient. Linearity, sensitivity, limit of quantification (LOQ) and limit of detection (LOD) were assayed by analyzing the calibration curves [89,90]. Repeatability was calculated as relative standard deviation at 10 μg·L^−1^ level, <1%; and reproducibility as relative standard deviation at 70 μg·L^−1^, <0.5%.

Compound	r	R^2^	B	M	Linearity (μg·L^−1^)	Sensibility (μg·L^−1^)	LOD (μg·L^−1^)	LOQ (μg·L^−1^)
Melatonin	0.999224	0.998060	164299.4	82342.3	0.9803	0.207	0.51	1.69

**Table 2:** Summary of the *C. annuum* L. genes encoding enzymes participating in melatonin biosynthesis and the characteristics of their corresponding proteins, including amino acid length (aa), molecular mass (kDa), theoretical isoelectric point (pI), and predicted subcellular localization. *T5H*, *tryptamine 5-hydroxylase*. *SNAT*, *serotonin N-acetyltransferase*. *ASMT*, *N-acetylserotonin O-methyltransferase*. *COMT*, *caffeic acid O-methyltransferase*. *ASDAC*, *N-acetylserotonin deacetylase*. The genes expressed in fruit are indicated in blue.

Gene Name	Gen ID	Chr	Protein ID	Length (aa)	Mw (kDa)	pI	Subcellular Location
*CaT5H1*	107866002	3	XP_016567673.2	503	57.39	6.31	Plastid
*CaT5H2*	107866001	3	XP_016567671.1	505	57.32	9.11	Plastid
*CaT5H3*	124898987	5	XP_047269291.1	522	59.19	5.86	Nucleus/Plastid
*CaT5H4*	107874621	6	XP_016576869.2	504	57.54	8.03	Plastid
*CaSNAT1*	107845856	10	XP_016545838.1	250	27.72	5.46	Plastid
*CaSNAT2*	107852754	11	XP_016553285.2	200	22.09	9.88	Plastid
*CaASMT1*	107880045	8	XP_016582450.1	363	40.35	5.58	Cytoskeleton
*CaASMT2*	107880046	8	XP_016582451.2	359	39.38	5.65	Cytoplasm
*CaASMT3*	107840901	Unplaced	XP_047259902.1	375	41.26	5.44	Cytoskeleton
*CaCOMT1*	107862991	3	NP_001311774.1	359	39.35	5.50	Plastid
*CaCOMT2*	124893919	Unplaced	XP_047260702.1	359	39.53	5.63	Plastid
*CaASDAC*	107867869	4	XP_047267820.1	444	48.21	6.78	Plastid

## Data Availability

The data presented in this study are available on request from the corresponding author. Sequence Read Archive (SRA) data remains confidential and will be released to the public upon project completion.

## Supplementary Materials

The following supporting information can be downloaded at https://www.mdpi.com/article/10.3390/antiox15040503/s1.
