# Supplementary material for: Identification and Regulation of Melatonin Biosynthetic Genes in Sweet Pepper During Ripening and Melatonin Treatment
Source: Antioxidants (Basel). 2026 Apr 17;15(4):503. doi: 10.3390/antiox15040503 (PMC13113004; doi:10.3390/antiox15040503)
Supplement: Supplementary file 1 [file antioxidants-15-00503-s001.zip › antioxidants-4207394-supplementary.pdf]

Supplementary Table S1. List of the different species used for the phylogenetic analysis of tryptamine 5-hydroxylase (T5H) proteins.

Plant specie	T5H Protein ID	Reference
Pepper (*Capsicum annuum* L.)	CaT5H1 (XP_016567673.2)<br>CaT5H2 (XP_016567671.1)<br>CaT5H3 (XP_047269291.1)<br>CaT5H4 (XP_016576869.2)	This study
Rice (*Oryza sativa* L.)	OsT5H1 (NP_001410381.1)	[13]
Tomato (*Solanum lycopersicum* L.)	SlT5H1 (NP_001234847.2)	[18]
Arabidopsis thaliana	AtT5H1 (NP_189261.1)<br>AtT5H2 (NP_172770.1)	[50]<br>[51]
Potato (*Solanum tuberosum*)	StT5H1 (XP_006359799.1)	[52]
Grape (*Vitis vinífera*)	VvT5H1 (XP_002276558.4)	[53]
Tobaccoo (*Nicotiana tabacum*)	NtT5H1 (XP_016442246.1)	[54]
Alfalfa (*Medicago truncatula*)	MtT5H1 (XP_003601148.2)	[55]
Apple (*Malus domestica*)	MdT5H1 (XP_008387476.2)	[56]
Soybean (*Glycine max*)	GmT5H1 (NP_001239692.1)	[57]
Cassava (*Manihot esculenta*)	MeT5H1 (XP_043804872.1)	[58]
Maiz (*Zea mays*)	ZmT5H1 (XP_008662542.1)	[59]
Orange (Citrus sinensis)	CsT5H1 (XP_015387424.1)	[60]
Sweet cherry (*Prunus avium*)	PaT5H1 (XP_021807680.1)	[61]

Supplementary Table S2. List of the different species used for the phylogenetic analysis of serotonin N-acetyltransferase (SNAT) proteins.

**Plant specie**	**SNAT Protein ID**	**Reference**
Pepper (*Capsicum annuum* L.)	**CaSNAT1** (XP_016545838.1)<br>**CaSNAT2** (XP_016553285.2)	This study
Rice (*Oryza sativa* L.)	OsSNAT1 (NP_001389503.1)<br>OsSNAT2 (NP_001409299.1)	[14]<br>[62]
Arabidopsis thaliana	AtSNAT1 (NP_173946.1)	[62]
Poplar (*Populus trichocarca*)	PtSNAT1 (XP_002312367.2)	[62]
Grape (*Vitis vinífera*)	VvSNAT1 (XP_002271276.1)	[62]
Sorghum (*Sorghum bicolor*)	BdSNAT1 (XP_003571288.1)	[62]
Barley (*Hordeum vulgare* subsp. vulgare)	HvSNAT1 (XP_044957652.1)	[62]
Tomato (*Solanum lycopersicum* L.)	SlSNAT1 (XP_004249035.1)<br>SlSNAT2 (XP_004238988.1)	[21]
Tobacco (*Nicotiana tabacum*)	NtSNAT1 (XP_016455489.1)<br>NtSNAT2 (XP_016501006.1)	[54]
Cassava (*Manihot esculenta*)	MeSNAT1 (XP_021621805.1)<br>MeSNAT2 (XP_021633373.1)	[58]
Potato (*Solanum tuberosum* L.)	StSNAT1 (XP_006362337.1)<br>StSNAT2 (XP_006348605.1)	[52]
Apple (*Malus domestica* Borkh.)	MdSNAT1 (XP_008388388.2)<br>MdSNAT2 (XP_008389353.2)	[56]
Strawberry (*Fragaria vesca*)	FvSNAT1 (XP_004302517.1)<br>FvSNAT2 (XP_004298329.1)	[63]
Wheat (*Triticum aestivum*)	TaSNAT1 (XP_044412662.1)<br>TaSNAT2 (XP_044422891.1)	[64]
Orange (*Citrus sinensis* L.)	CsSNAT1 (XP_006481240.2)<br>CsSNAT2 (XP_006484294.2)	[60]
Maize (*Zea mays*)	ZmSNAT1 (NP_001338882.1)<br>ZmSNAT2 (NP_001143948.2)	[65]
Sweet cherry (*Prunus avium*)	PaSNAT1 (XP_021815082.1)	[61]
Soybean (*Glycine max*)	GmSNAT1 (XP_006602732.1)<br>GmSNAT2 (XP_003551622.2)	[66]
Peach (*Prunus persica*)	PpSNAT1 (XP_007205871.2)<br>PpSNAT2 (XP_007225898.1)	[67]

Supplementary Table S3. List of the different species used for the phylogenetic analysis of N-acetylserotonin O-methyltransferase (ASMT) proteins.

**Plant specie**	**ASMT Protein ID**	**Reference**
Pepper (*Capsicum annuum L.*)	CaASMT1 (XP_016582450.1)<br>CaASMT2 (XP_016582451.2)<br>CaASMT3 (XP_047259902.1)	This study
Rice (*Oryza sativa* L.)	OsASMT1 (NP_001409660.1)<br>OsASMT2 (XP_015613151.1)<br>OsASMT3 (NP_001409903.1)	[13]<br>[68]
Arabidopsis thaliana	AtASMT1(NP_195242.1)	[13]
Oat (*Avena strigosa*)	AsASMT1 (AFU52936.1)	[13]
Wheat (*Triticum dicoccoide*)	TdASMT1(XP_037422451.1)	[13]
Foxtail millet (*Setaria italica*)	SiASMT1 (XP_004973447.1)	[13]
Sorghum (*Sorghum bicolor*)	SbASMT1 (XP_002441380.2)	[13]
Grape (*Vitis vinífera*)	VvASMT1 (XP_002278092.1)	[13]
Barley (*Hordeum vulgare subsp. vulgare*)	HvASMT1 (BAK00281.1)	[13]
China rose (*Rosa chinensis*)	RcASMT1 (XP_024185755)	[13]
Cacao (*Theobroma cacao*)	TcASMT1 (XP_007043718.2)	[13]
Cucumber (*Cucumis sativus*)	CsASMT1 (XP_004151735.2)	[13]
Tangerine (*Citrus x clementina*)	CcASMT1 (XP_006446728.1)	[13]
Plum (*Prunus mume*)	PmASMT1 (XP_008245137.1)	[13]
Mulberry (*Morus notabilis*)	MnASMT1 (EXC30549.1)	[13]
Potato (*Solanum tuberosum* L.)	StASMT1 (XP_006366298.1)	[13]
Soybean (*Glycine max*)	GmASMT1 (XP_003536188.1)	[13]
Alfalfa (*Medicago truncatula*)	MtASMT1 (XP_003615967.1)	[13]
Common bean (*Phaseolus vulgaris*)	PvASMT1 (XP_007142077.1)	[13]
Pear (*Pyrus pyrifolia*)	PpASMT1 (BAA86059.1)	[13]
California poppy (*Eschscholzia californica*)	EcASMT1 (BAM37634.1)	[13]
Pine (*Pinus taeda*)	PtASMT1 (AAC49708.1)	[13]
Apple (*Malus zumi*)	MzASMT1 (AIY62760.1)	[69]
Tomato (*Solanum lycopersicum* L.)	SlASMT1 (Solyc03g080180.2.1)	[70]

Supplementary Table S4. List of the different species used for the phylogenetic analysis of caffeic acid O-methyltransferase (COMT) proteins.

Plant specie	COMT protein ID	Reference
Pepper (*Capsicum annuum L.*)	CaCOMT1 (NP_001311774.1)<br>CaCOMT2 (XP_047260702.1)	This study
Rice (*Oryza sativa*)	OsCOMT1 (NP_001390411.1)	[17]
Arabidopsis thaliana	AtCOMT1 (NP_200227.1)	[15]
Grape (*Vitis vinífera*)	VvCOMT1 (NP_001268100.11)	[71]
Model grass (*Brachypodium distachyon*)	BdCOMT1 (XP_003573470.1)	[72]
Tomato (*Solanum lycopersicum* L.)	SlCOMT1 (XP_004235028.1)	[73]
Tobacco (*Nicotiana tabacum*)	NtCOMT1 (XP_016465165.1)	[54]
Cassava (*Manihot esculenta*)	MeCOMT1 (XP_021627291.1)	[58]
Patato (Solanum tuberosum)	StCOMT1 (XP_015164331.1)	[52]
Apple (*Malus domestica*)	MdCOMT1 (XP_008347272.2)	[56]
Strawberry (*Fragaria vesca*)	FvCOMT1 (XP_004307899.1)	[63]
Alfalfa (*Medicago truncatula*)	MtCOMT1 (XP_003602396.1)	[55]
Cotton (*Gossypium hirsutum*)	GhCOMT1 (NP_001313943.1)	[74]
Orange (*Citrus sinensis*)	CsCOMT1 (XP_006478090.2)	[60]
Maize (*Zea mays*)	ZmCOMT1 (NP_001106047.1)	[65]
Sweet cherry (*Prunus avium*)	PaCOMT1 (XP_021822608.1)	[61]
Soybean (*Glycine max*)	GmCOMT1 (XP_003526767.1)	[66]
Peach (*Prunus persica*)	PpCOMT1 (XP_007218167.1)	[67]
Pea (*Pisum sativum*)	PsCOMT1 (XP_050877436.1)	[75]
Spinach (*Spinacia oleracea*)	SoCOMT1 (XP_021858404.1)	[76]
Lettuce (*Lactuca sativa*)	LaCOMT1 (XP_023771773.1)	[77]

Supplementary Table S5. List of the different species used for the phylogenetic analysis of N-acetylserotonin deacetylase (ASDAC) proteins.

Plant species	ASDAC protein ID	Reference
Pepper (*Capsicum annuum L.*)	**CaASDAC** (XP_047267820.1)	This study
Rice (*Oryza sativa*)	OsASDAC (XP_015618357.1)	[43]
Arabidopsis thaliana	AtASDAC (NP_567921.1)	[43]
Grape (*Vitis vinifera*)	VvASDAC (XP_002267516.1)	[53]
Tomato (*Solanum lycopersicum*)	SlASDAC (XP_004228472.1)	[79]
Tobacco (*Nicotiana tabacum*)	NtASDAC (XP_016447317.1)	[54]
Cassava (*Manihot esculenta*)	MeASDAC (XP_021631441.1)	[54]
Potato (*Solanum tuberosum*)	StASDAC (XP_006364494.1)	[52]
Apple (*Malus domestica*)	MdASDAC (XP_017178842.1)	[56]
Strawberry (*Fragaria vesca*)	FvASDAC (XP_004288101.1)	[63]
Wheat (*Triticum aestivum*)	TaASDAC (XP_044387692.1)	[64]
Orange (*Citrus sinensis*)	CsASDAC (XP_015384642.1)	[60]
Maize (*Zea mays*)	ZmASDAC (NP_001402758.1)	[79]
Sweet cherry (*Prunus avium*)	PaASDAC (XP_021807318.1)	[61]
Soybean (*Glycine max*)	GmASDAC (XP_014631275.1)	[66]
Peach (*Prunus persica*)	PpASDAC (XP_007211505.2)	[67]

Supplementary Table S6. Summary of the data obtained from sequencing the 12 cDNA libraries: Two independent replicates were conducted for each ripeness stage of pepper fruits, including two for Green, two for Red, and two for each breakpoint (BP) of the treatments with varying concentrations of melatonin (0, 20, 50, and 100 μM). The robustness of the transcriptomic profile is evidenced by a high-depth sequencing yield of 291,697,758 raw reads across the 12 cDNA libraries, maintaining an average of 83.10% useful reads and a high alignment rate to the transcriptome (93.23%), which ensures statistical power and internal consistency across all ripeness stages and melatonin treatments.

Ripeness Stages	Sample	Raw Reads	Trimmed Reads	% Useful Reads	% Aligned Reads to Transcriptome
**Green (G)**	1_1	23,402,352	19,219,743	82.13	93.01
	1_2	25,692,613	20,999,077	81.73	92.71
**Red (R)**	2_1	24,474,541	20,808,205	85.02	93.02
	2_2	23,958,860	19,933,483	83.20	93.23
**BP+0MEL**	3_1	24,844,537	21,046,770	84.71	93.30
	3_2	26,469,059	22,416,609	84.69	93.72
**BP+20MEL**	4_1	22,169,524	17,852,453	80.53	92.66
	4_2	22,048,684	18,045,499	81.84	93.25
**BP+50MEL**	5_1	24,127,512	19,792,048	82.03	93.43
	5_2	25,463,178	21,255,768	83.48	93.88
**BP+100MEL**	6_1	25,962,761	22,124,382	85.22	93.23
	6_2	23,084,137	18,910,387	81.92	92.85
**Total**	-	291,697,758	242,404,424	83.10	93.23

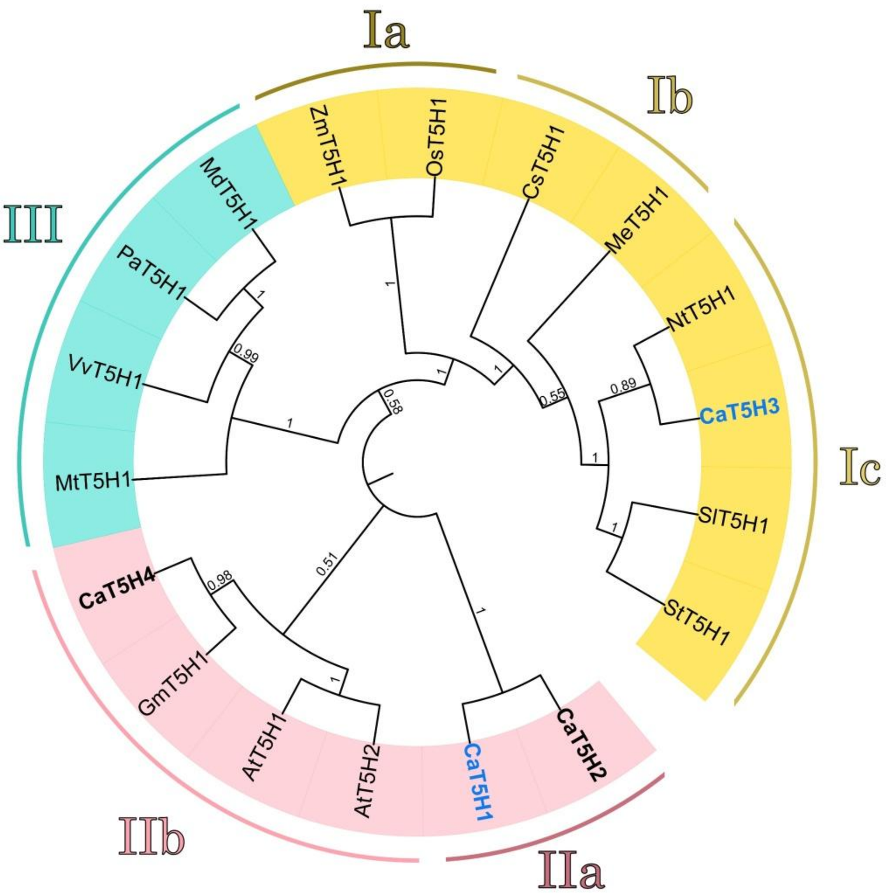

Figure S1. Phylogenetic relationships between tryptamine 5-hydroxylases (T5H) from different plant species. *Clusters (I-III) are displayed using different colors. Species abbreviations:* Ca (*Capsicum annuum L.*), Os (*Oryza sativa*), *Sl (Solanum lycopersicum),* At (*Arabidopsis thaliana*), St (*Solanum tuberosum*), Vv *(Vitis vinifera*), Nt (*Nicotiana tabacum*), Mt (*Medicago truncatula*), Md (*Malus domestica*), Gm (*Glycine max*), Me (*Manihot esculenta),* Zm (*Zea mays*), Cs (*Citru sinensis*), Pa (*Prunus avium*). CaT5Hs detected in pepper are in bold letters and those identified in the transcriptome of pepper fruit are highlighted in blue.

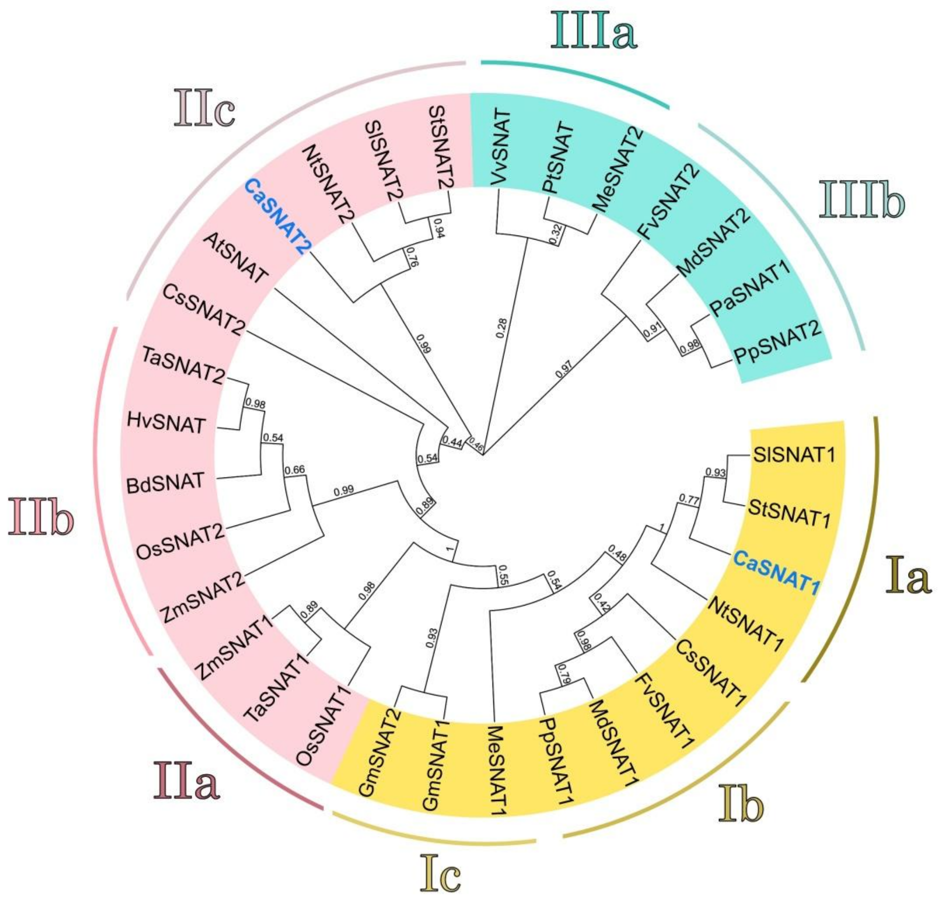

Figure S2. Phylogenetic relationships between serotonin N-acetyltransferase (SNAT) from different plant species (identifiers are listed in Supplementary Table S2). Clusters (I-III) are displayed using different colors. Apple (*Malus domestica*, Md), *Arabidopsis thaliana* (At), barley (*Hordeum vulgare*, Hv), cassava (*Manihot esculenta*, Me), grape (*Vitis vinífera*, Vv), maize (*Zea mays*, Zm), orange (*Citrus sinensis*, Cs), peach (*Prunus persica*, Pp), pepper (*Capsicum annuum*, Ca), poplar (*Populus trichocarca,* Pt), potato (*Solanum tuberosum*, St), rice (*Oryza sativa*. Os), sorghum (*Sorghum bicolor*, Sb), soybean (*Glycine max*, Gm), strawberry (*Fragaria vesca*, Fv), sweet cherry (*Prunus avium*, Pa), tobacco (*Nicotiana tabacum*, Nt), tomato (*Solanum lycopersicum*, Sl), and wheat (*Triticum aestivum*). CaSNATs detected in pepper are highlighted in blue.

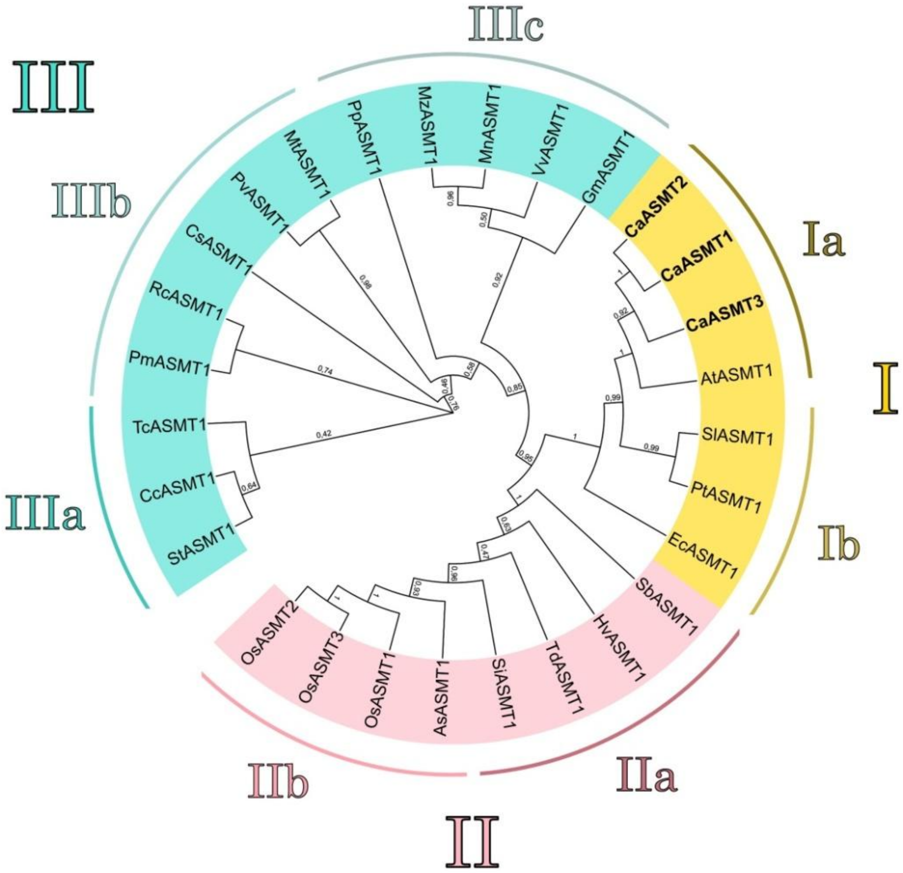

Figure S3. Phylogenetic relationships between N-acetylserotonin O-methyltransferase (ASMT) from different plant species (identifiers are listed in Supplementary Table S3). Clusters (I-III) are displayed using different colors. Alfalfa (Medicago truncatula, Mt), apple (Malus zumi, Mz), Arabidopsis thaliana (At), barley (Hordeum vulgare, Hv), cacao (Theobroma cacao, Tc), california poppy (Eschscholzia californica, Ec), china rose (Rosa chinensis, Rc), common bean (Phaseolus vulgaris, Pv), cucumber (Cucumis sativus, Cs), foxtail millet (Setaria italic, Si), grape (Vitis vinífera, Vv), mulberry (Morus notabilis, Mn), oat (Avena strigose, As), pear (Pyrus pyrifolia, Pp), pepper (Capsicum annuum, Ca), pine (Pinus taeda, Pt), plum (Prunus mume), potato (Solanum tuberosum L.), rice (Oryza sativa, Os), sorghum (Sorghum bicolor, Sb), soybean (Glycine max, Gm), tangerine (Citrus x clementina, Cc), tomato (Solanum lycopersicum, Tl), and wheat (Triticum dicoccoide, Td).

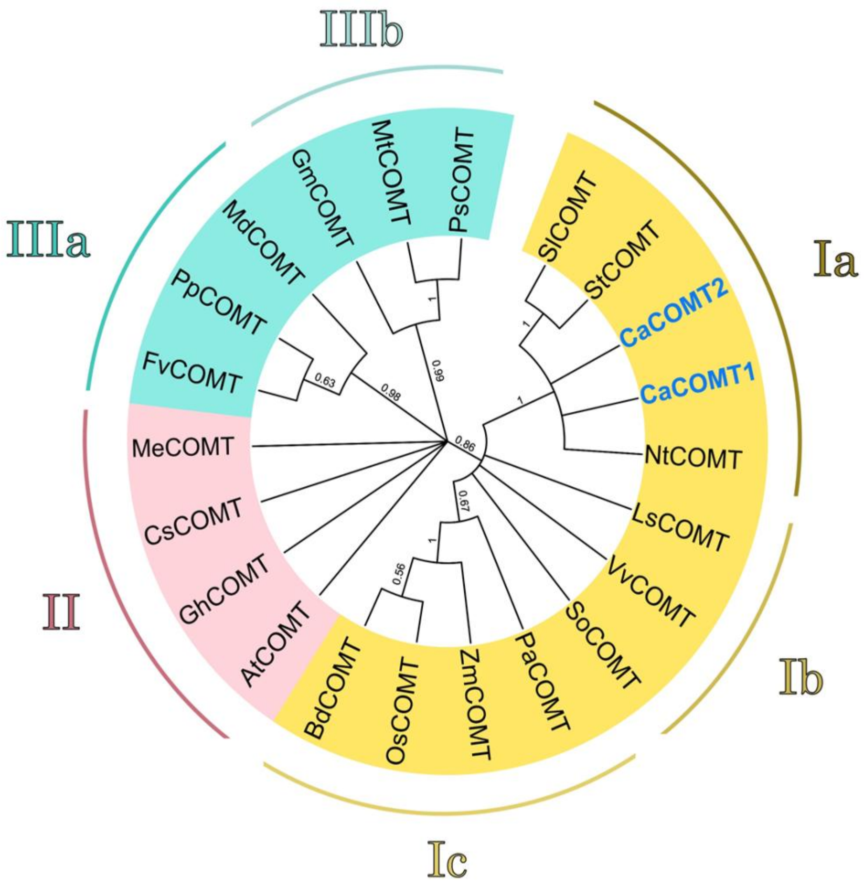

Figure S4. Phylogenetic relationships between Caffeic acid O-methyltransferase (COMT) from different plant species (identifiers are listed in Supplementary Table S4). Clusters (I-III) are displayed using different colors. Alfalfa (*Medicago truncatula*, Mt), apple (*Malus domestica*, Md), *Arabidopsis thaliana* (At), cassava (*Manihot esculenta*, Me), cotton (*Gossypium hirsutum*, Gh), grape (*Vitis vinífera*, Vv), lettuce (*Lactuca sativa*, Ls), maize (*Zea mays*, Zm), model grass (*Brachypodium distachyon*, Bd), orange (*Citrus sinensis*, Cs), potato (*Solanum tuberosum*, St), pea (*Pisum sativum*, Ps), peach (*Prunus persica*, Pp), pepper (*Capsicum annuum*, Ca), rice (*Oryza sativa*, Os), soybean (*Glycine max*, Gm), spinach (*Spinacia oleracea*, So), strawberry (*Fragaria vesca*, Fv), sweet cherry (Prunus avium, Pa), tobacco (*Nicotiana tabacum*, Nt), and tomato (*Solanum lycopersicum*, Sl). CaCOMTs detected in pepper are highlighted in blue.

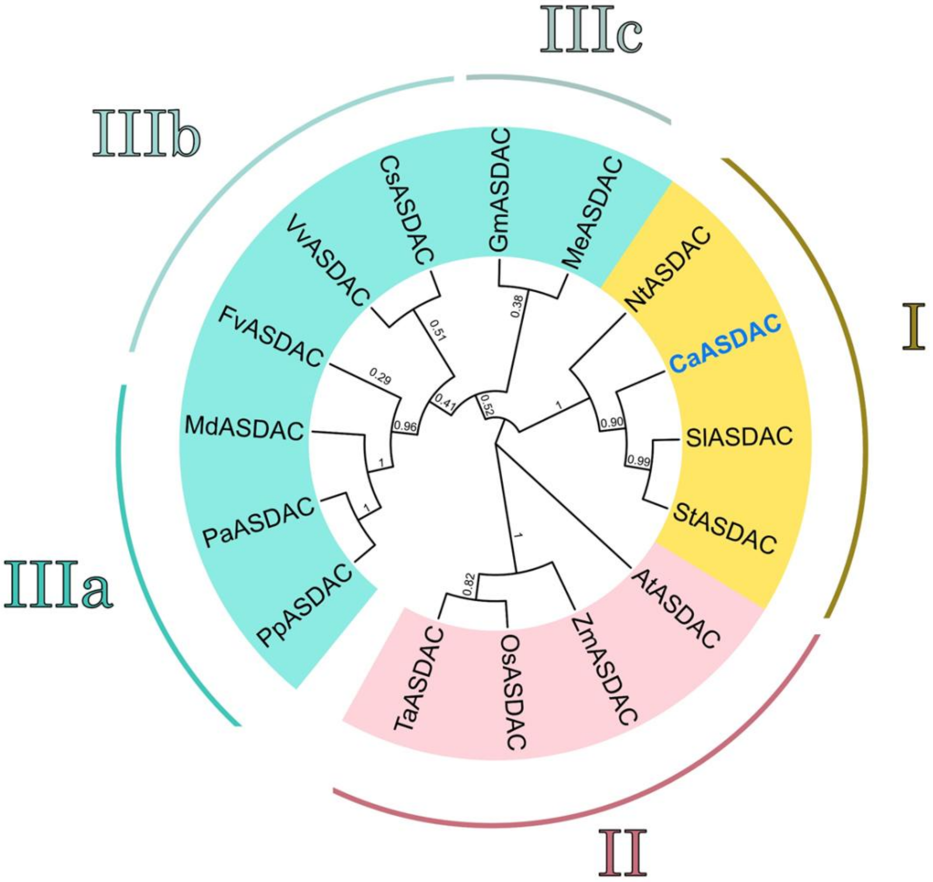

Figure S5. Phylogenetic relationships between N-acetylserotonin deacetylase (ASDAC) from different plant species (identifiers are listed in Supplementary Table S5). Clusters (I-III) are displayed using different colors. Apple (Malus domestica, Md), Arabidopsis thaliana (At), cassava (Manihot esculenta, Me), grape (Vitis vinifera, Vv), maize (Zea mays, Zm), orange (Citrus sinensis, Cs), peach (Prunus persica, Pp), pepper (Capsicum annuum, Ca), potato (Solanum tuberosum, Sl), rice (Oryza sativa, Os), soybean (Glycine max, Gm), strawberry (Fragaria vesca, Fv), sweet cherry (Prunus avium, Pa), tobacco (Nicotiana tabacum, Nt), tomato (Solanum lycopersicum, Sl), and wheat (Triticum aestivum, Ta). CaASDACs detected in pepper are highlighted in blue.
